# Defined domains and cleavage determine the diverse functions of piscine myocarditis virus p33 protein

**DOI:** 10.3389/fmicb.2025.1633241

**Published:** 2025-09-01

**Authors:** Racheal Amono, Snøa A. T. N. Fredlund, Morgane Chesnais, Bernd Thiede, Turhan Markussen, Øystein Evensen, Aase B. Mikalsen

**Affiliations:** ^1^Department of Paraclinical Sciences, Faculty of Veterinary Medicine, Norwegian University of Life Sciences, Ås, Norway; ^2^Department of Biosciences, University of Oslo, Oslo, Norway

**Keywords:** PMCV, *Pistolviridae*, dsRNA virus, p33 protein, infection mechanisms, CMS

## Abstract

Piscine myocarditis virus (PMCV) causes chronic, necrotizing myocarditis in Atlantic salmon. Originally, PMCV was identified based on its genetic homology and genomic organization, indicating a relationship to viruses of the *Ghabrivirales* order, specifically the former *Totiviridae* family, whose members predominantly infect fungi or protozoans and lack an extracellular life cycle stage. However, PMCV was the first virus of this order found to infect a vertebrate host. Since then, other piscine viruses and viruses infecting terrestrial and aquatic arthropods have been described and recently assigned to new virus families within the order. PMCV is now classified in *Pistolviridae*. All these viruses infecting multicellular hosts encode proteins that are believed to be involved in extracellular transmission. In PMCV, this relates to a protein of size 33.4 kDa (p33) encoded by a unique third open reading frame. To investigate its characteristics and role, we expressed various recombinant variants of p33 in cultured cells. Our results demonstrate that p33 expression induces a cytotoxic phenotype in transfected cells. The full-length protein undergoes processing into smaller peptide variants. Previous *in silico* analysis predicted an N-terminal chemokine-like domain, and our present results show that this domain is secreted as peptides capable of inducing cytotoxicity when expressed alone. The C-terminal region includes sequence characteristics of a small hydrophobic domain, which appears crucial for the correct processing of the full-length protein into N- and C-terminal peptides and directing the C-terminal peptides to a high membrane concentration. Investigations into p33 function could elucidate how PMCV achieves extracellular transmission, a mechanism that may be conserved among viruses of *Pistolviridae*. The findings in this study provide evidence that p33 has structural and functional characteristics of a protein adapted to facilitate host cell membrane interaction and cell lysis, potentially enabling extracellular viral release. These insights may provide evolutionary evidence that pistolviruses have acquired the uncommon trait of viral transmission within the order *Ghabrivirales*, broadening our understanding of virus–host adaptation in vertebrates.

## 1 Introduction

Piscine myocarditis virus (PMCV) infects Atlantic salmon (*Salmo salar* L.), causing cardiomyopathy syndrome (CMS), a disease characterized by necrotizing myocarditis (Haugland et al., 2011). Clinical cases of CMS have been reported in aquaculture operations across Norway, the Faroe Islands, Scotland, Ireland, and potentially Canada (Rodger and Turnbull, 2000; Brocklebank and Raverty, 2002; Rodger et al., 2014). The virus was first described as sharing genomic characteristics with viruses of the family *Totiviridae*, order *Ghabrivirales*, which includes viruses that persistently infect protozoan parasites and fungi. Over the last decade, more complex viruses with genetic similarities to *Totiviridae* have been described from terrestrial and aquatic arthropods, bats, planarians, and piscine species, including PMCV (Wu et al., 2010; Zhai et al., 2010; Isawa et al., 2011; Shi et al., 2016; Prasad et al., 2017; Burrows et al., 2020; Bačnik et al., 2021; Kohl et al., 2021; Sandlund et al., 2021; Louboutin et al., 2023; Couto et al., 2024). PMCV was the first “toti-like” virus found to infect a vertebrate host and forms a phylogenetic cluster with the majority of the later-described piscine viruses. The order *Ghabrivirales* was recently reorganized, and PMCV, along with three additional piscine viruses characterized by extra open reading frame(s) (ORF) at the 3′ end of the genome, was assigned to a new virus family named *Pistolviridae* (Current ICTV Taxonomy Release, 2023). The three additional viruses are golden shiner toti-like virus 1 [GSTLV-1, from golden shiner (*Notemigonus crysoleucas*)], *Cyclopterus lumpus* toti-like virus [CLuTLV, from lumpsucker (*Cyclopterus lumpus*)], and common carp toti-like virus [CCTLV-1, from common carp (*Cyprinus carpio*)] (Mor and Phelps, 2016; Sandlund et al., 2021). It is also reasonable to expect that the recently described sea bass toti-like virus (SBTLV) from European sea bass (*Dicentrarchus labrax*) (Louboutin et al., 2023) will be included in *Pistolviridae*.

The genomes of viruses in the order *Ghabrivirales,* with a few exceptions, are composed of a single molecule of double-stranded (ds) RNA containing two open reading frames (ORFs) that encode a capsid protein and an RNA-dependent RNA polymerase (RdRp), organized with a ribosomal frameshift that may result in the expression of a fused capsid-RdRp protein (Current ICTV Taxonomy Release, 2023). The genome organization of PMCV capsid and RdRp ORFs is very similar, including a predicted −1 ribosomal frameshift. The 3′ end of the genome has a unique third ORF (ORF3) encoding a protein of 302 amino acids with a predicted molecular mass of 33.4 kDa. No significant sequence homology has been found at the nucleotide or amino acid level with viruses of the same order, other viruses, or proteins. However, a putative chemokine superfamily motif has been identified in the N-terminus of the protein (Haugland et al., 2011). The piscine GSTLV-1 and CCTLV-1 also include a similar 3′ end ORF3. These viruses share 76 and 52% pairwise amino acid identities in the capsid and 70 and 71% in the RdRp with PMCV, respectively. In contrast, the homologies of ORF3-encoded proteins are insignificant, with pairwise amino acid sequence identities of only 14–21%. Nevertheless, the ORF3-encoded proteins from all three viruses share characteristics of predicted domains, including N-terminal signal sequences, followed by the presence of the putative chemokine superfamily motif and a hydrophobic, likely transmembrane domain in the C-terminal half of the protein sequence (Sandlund et al., 2021).

The low similarities between these ORF3-encoded proteins and their low homology to any known proteins have raised several questions regarding the function of the ORF3-encoded protein in the virus’ pathogenesis and/or replication. The viruses assigned to the *Ghabrivirales* order are generally transmitted between cells during cell division, sporogenesis, or cell fusion (Current ICTV Taxonomy Release, 2023). In contrast, viruses of the families *Giardiaviridae* (protozoan hosts), *Artiviridae* (arthropod hosts), and *Pistolviridae* are known or presumed to transmit extracellularly. Additional protein-coding sequences have been suggested to encompass all or some of their cell entry and exit machinery, which for the artiviruses and pistolviruses also include more advanced infection routes in multicellular organisms (Miller et al., 1988; Poulos et al., 2006; Haugland et al., 2011; Nibert and Takagi, 2013; Dantas et al., 2016). This study was undertaken to characterize the PMCV ORF3-encoded protein, named p33 based on the calculated mass of 33.4 kDa, to better understand the protein’s role in virus replication.

## 2 Materials and methods

### 2.1 *In silico* analyses

All *in silico* analyses were performed on sequences from the PMCV reference isolate AL V-708 (GenBank accession number HQ339954). The prediction of a signal sequence, hydrophobic/transmembrane regions, protein secondary structures, and three-dimensional (3D) structure modeling of the chemokine-like domain has been presented previously (Sandlund et al., 2021). The 3D modeling of the chemokine-like domain was repeated using the Phyre2 web portal for protein modeling, prediction, and analysis (Kelley et al., 2015), which confirmed the previously described 3D model. Sites for N-glycosylation and disulfide bonds were predicted using the NetNglyc 1.1 server (Gupta and Brunak, 2002) and DISULFIND (Ceroni et al., 2006), respectively. The Compute pI/Mw tool (Expasy) calculated the molecular weights of proteins and peptides.

### 2.2 PMCV reference isolate and field material

The major *in vitro* recombinant expression experiments were performed using the PMCV AL V-708 isolate (GenBank accession number HQ339954; Haugland et al., 2011). This is the first PMCV isolate described and is hereafter referred to as the reference isolate. In addition, from an in-house sample depository, 11 field PMCV strains, including sequence characteristics data (Amono et al., 2024), were chosen for recombinant expression studies (referred to as wt1-11).

### 2.3 Cell lines

The standard fish cell lines used for recombinant expression of the proteins were CHH-1, originating from chum salmon (*Oncorhynchus keta*) heart tissue (Lannan et al., 1984), and Epithelioma papulosum cyprini (EPC) cells, which originate from the skin tissue of Fathead minnow (*Pimephales promelas*) (ATCC). The cells were propagated at 20°C in Leibovitz’s L15 medium (Invitrogen) supplemented with 10% fetal bovine serum (FBS; Invitrogen). Additionally, some experiments used the chinook salmon embryo (CHSE) cells, originating from the embryo tissue of Chinook salmon (*Oncorhynchus tshawytscha*). CHSE cells were maintained under conditions identical to those of CHH-1. All cell media were supplemented with 50 mg/mL gentamicin (Invitrogen), unless otherwise stated.

### 2.4 Construction of plasmid vectors for expression from PMCV ORF1, ORF2, and ORF3

The full-length genome of the PMCV reference isolate was synthesized using a commercial service (Aldevron) and delivered as a plasmid with a pUC57 backbone. The resulting rPMCV-pUC57 plasmid construct was used as a template in subsequent PCR amplifications of the three ORFs.

Each ORF was amplified using the DyNAzyme EXT DNA polymerase kit (Thermo Scientific) and primers designed to add 5’ *Xho*I and 3’ *Hind*III restriction enzyme sites while excluding the stop codons for cloning into pmaxFP-Green-N (Amaxa Biosystems) for recombinant expression with the green fluorescent protein (GFP) attached to their C-terminal end (Supplementary Table S1, plasmids pORF1green, pORF2green, and pORF3green). In short, the resulting PCR products were purified using standard procedures and commercial kits and subsequently ligated into the pCR 2.1 vector using a Topo TA cloning kit (Invitrogen), followed by transformation into competent OneShot TOP10 bacterial cells (Invitrogen). The ORFs were then subcloned using the 5’ *Xho*I and 3’ *Hind*III restriction enzyme sites into the expression vectors using conventional cloning procedures and kits with NEB 10-beta Competent *E. coli* (New England Biolabs). Bacteria from all transformations were seeded onto LB agar plates with 100 μg/mL ampicillin or 50 μg/mL kanamycin, as applicable for each plasmid vector backbone, and grown at 30 or 37°C for 20–24 h. ORF3 amplicons encoding p33 wt1-11, generated from the available cDNA from previous analyses (Amono et al., 2024), were cloned into pmaxFP-Green-N similarly.

ORF3, including the stop codon, was also amplified using cDNA available in the lab from heart tissue sampled from Atlantic salmon experimentally infected with PMCV AL V-708 (Haugland et al., 2011). The ORF3 amplicon was cloned into pmaxFP-Green-C (Amaxa Biosystems) using 5’ *Xho*I and 3’*Hind*III as described above, for the expression of the p33 reference with an N-terminal GFP tag (Supplementary Table S1, plasmid pGreenORF3).

Selected colonies representing all plasmid variants were cultured in LB medium with appropriate antibiotics at 37°C for 20–24 h before the plasmids were purified using commercial kits. Purified plasmids were quantified, and purity was analyzed using the OD260/280 ratio on a NanoDrop ND-1000 (NanoDrop Technologies). PMCV ORF inserts in the plasmid expression vectors were verified by sequencing prior to use through a commercial sequencing service (Eurofins). An overview of all plasmid constructs, including the sequence origin of PMCV inserts, primers used, and the name of the final product, is given in Supplementary Table S1.

### 2.5 *In vitro* mutagenesis of plasmid expression vectors for functional characterization of selected PMCV p33 variants

A vector expressing p33 with the C-terminal GFP tag exchanged for a His tag (6x Histidine) was constructed by amplifying a plasmid copy that excluded the GFP tag while adding six repetitions of codons for Histidine, using the Phusion Site-Directed Mutagenesis Kit (Thermo Scientific), following the manufacturer’s procedure and the primers described in Supplementary Table S1. A vector expressing p33 with a Flag tag (DYKDDDDK) at the N-terminal end, in addition to an intact C-terminal GFP tag, was constructed using similar procedures (Supplementary Table S1). The vector was designed to add the Flag tag between residues 22 and 23, i.e., subsequent to the predicted signal sequence (residues 1–20), to preserve the function of the signal sequence and ensure the tag remains on the resulting N-terminal p33 end after the release of the signal sequence.

Vectors expressing recombinant p33 with deletions or mutations while retaining the C-terminal GFP tag were constructed using similar procedures (Supplementary Table S1). In brief, PCR amplification was performed using 250 pg of pORF3green as the template and primers, including the desired mutation/addition in one of the primers and/or excluding a sequence region by positioning the primer set (Supplementary Table S1).

The amplified linear plasmid amplicons were circularized by ligation. Subsequently, NEB 10-beta competent *E. coli* were transformed with the circularized products following the manufacturer’s procedures and seeded on LB agar plates as described earlier.

### 2.6 Transfection of cultured fish cells for recombinant protein expression

Cultured fish cells were transfected by electroporation using the Neon™ Transfection system (Invitrogen) with a 10-μl or 100-μl tip, following the manufacturer’s recommendations. A mixture of cells and plasmids was subjected to electroporation with a single pulse at 1200 V and a pulse width of 40 ms. Each 10 μL transfection included 2 × 10^5^ cells combined with 1 μg of plasmid, and the cells were seeded into one well of a 24-well multidish, distributed over five wells in a 96-well multidish, or over two wells in eight-well chamber glass slides (BD Falcon). Each 100 μL transfection included 1.5× 10^6^ cells and 10 μg of plasmid, which was seeded into one well of a 6-well multidish after electroporation. All electroporated cells were grown in standard culture medium as described above, excluding antibiotics.

Chemical transfection was performed using the X-tremeGENE HP DNA reagent (Roche). Either 0.4 or 2 μg of plasmid was mixed with 3 μL of X-tremeGENE HP Transfection Reagent and incubated for 5 min at room temperature before adding 100 μL of L15 medium. The mixture was further incubated for 1 h at room temperature. Subsequently, the mixture was transferred to cells at approximately 70% confluency in standard cell culture medium as described above, using 20 μL of the mix per well in a 24-well multidish.

All transfected cell lines were incubated at the standard temperature for each cell line. The results were analyzed and imaged using a phase contrast microscope (Olympus IX81) with fluorescence capabilities. All p33 variants were expressed and examined by microscopy for at least 5 days post-transfection (dpt) in repeated assays with consistent results.

### 2.7 Fluorescent staining of recombinant expressed PMCV p33 with His-tag and of cellular compartments *in vitro*

Expression of His-tagged proteins in cultured cells was detected using immunofluorescent staining. The cells were fixed in 3.7 or 4% paraformaldehyde in PBS for 10–20 min and permeabilized using Triton X-100 (Thermo Scientific) before being subjected to Anti-6X His tag® antibody (Abcam) as the primary antibody and Alexa Fluor® 488 goat anti-mouse IgG2b as the secondary antibody. Cell membranes of live cells were stained using a 1:10^4^ dilution of CellMask™ Orange plasma membrane stain (Molecular Probes) in L15 without additives, followed by fixation in ice-cold 3.7% formaldehyde with the cells on ice. Nuclei were stained in pre-fixed cells using a 10-min incubation in 1 μM Hoechst 33342. The Hoechst stain was part of the Image-IT™ Live kit (Invitrogen) and was also combined with 2 μg/mL of red-fluorescent Alexa Fluor® 594 wheat germ agglutinin (WGA) from the kit, staining sialic acid and N-acetylglucosaminyl residues of plasma and trans-Golgi membranes. All dilutions of stains and washing of cell monolayers were performed in PBS, and all incubations after fixation were performed at room temperature. Analysis and imaging were conducted using a phase contrast microscope (Olympus IX81) with fluorescence capabilities while in PBS. For confocal microscopy, the transfected cells in eight-well chamber glass slides were fixed and stained as described above and mounted with Vectashield antifade mounting medium (Vector Laboratories) before analysis using a Leica SP8 Stellaris confocal microscope (Leica).

### 2.8 Apoptosis assays

Cell death mechanisms were investigated using two assays. First, DNA fragmentation as an indication of apoptosis was assessed in both EPC and CHH-1 cells expressing the recombinant protein p33 with a C-terminal GFP tag, harvested at 2 and 3 days post-transfection (dpt). Cells expressing GFP only were included as a negative control, while cells treated with Staurosporine (Biotinum) at a final concentration of 0.5 μM served as a positive control. According to the kit protocol, the cells were harvested from 6-well multidishes, and cellular DNA was isolated using the Apoptotic DNA ladder kit (Roche) following the manufacturer’s recommendations, which included the final degradation of any contaminating RNA. An aliquot of each DNA sample was separated by gel electrophoresis in a 1% agarose gel to reveal any DNA fragmentation.

Second, a fluorescence-staining assay was performed to distinguish the compacted state of the chromatin in the nuclei of apoptotic cells from the nuclei of necrotic (permeable) cells. This assay was conducted on CHH-1 cells expressing the recombinant protein p33 after transfection via electroporation. Cells expressing the PMCV capsid with a C-terminal GFP tag and mock (no plasmid) transfected cells were used as controls. Cells were cultured in a 96-well multidish, washed carefully in L15 medium, and subjected to a staining reagent mix containing 1 μM Hoechst 33342 and 1 μg/mL propidium iodide, followed by a 20-min incubation at room temperature and another wash. Analysis and imaging were performed in L15 medium without further fixation, using a phase contrast microscope (Olympus IX81) with fluorescence capabilities. The blue-fluorescent Hoechst 33342 dye stains the condensed chromatin of apoptotic cells more brightly than the chromatin in the nuclei of normal cells. The red-fluorescent propidium iodide dye stains only the nuclei of dead cells with compromised membranes. Cells in parallel wells were stained at 1, 2, 3, and 6 dpt.

### 2.9 Lactate dehydrogenase (LDH) assay for quantification of cell death *in vitro*

Cell death was measured by analyzing the levels of the cytosolic enzyme lactate dehydrogenase (LDH) released into the surrounding cell culture medium after damage to the plasma membrane. As described above, EPC cells were transfected with various expression plasmids for p33 variants using the Neon™ Transfection system (Invitrogen) with a 100-μl tip, including 1 × 10^6^ cells and 10 μg plasmid for electroporation. Electroporated cells were transferred to one well in a 12-well multidish. Triplicate wells were prepared for each expression plasmid variant by repeating the procedure. After 3.5 h of incubation, the cell culture medium, including non-attached cells, was removed and replaced with 1.2 mL of fresh L15 medium containing 10% FBS. Aliquots of 75 μL of the medium were harvested per well at 5, 28, 68, and 96 h post-transfection (hpt) and centrifuged at 1200 rpm for 5 min to remove any cells or cell debris, then stored at −20°C until analysis. The LDH levels were measured using 50 μL of each harvested medium sample with the CyQUANT™ LDH Cytotoxicity assay kit (Invitrogen) following the manufacturer’s recommendations. Absorbance in each reaction well was measured at 490 nm and 680 nm using a Spark® Multimode Microplate Reader (Tecan). LDH levels were calculated by subtracting the background signal at 680 nm from the 490 nm signal.

### 2.10 Harvesting of protein from cells and supernatant, immunoprecipitation, SDS-PAGE and Western blot

Samples for SDS-PAGE and Western blot analyses were prepared from transfected cells cultured in six-well multidishes. The cells in each well were lysed by incubation in 250 μL of CelLytic M (Sigma) for 15 min at room temperature with gentle shaking. The harvested lysate was centrifuged for 20 min at 16,000x g at 4°C to remove cell debris. A total of 22.5 μL of the lysate supernatant was mixed with 6 μL of 5x Loading Buffer and 1.5 μL of 20x Reducing Agent (Fermentas) and denatured at 99°C for 5 min, followed by immediate cooling on ice. To detect Flag-tagged proteins in the supernatant, the cell cultures for analysis were grown with 50% lower medium volume to increase the concentration of secreted protein products, followed by immunoprecipitation using Dynabeads® Protein G (Novex) according to the manufacturer’s instructions. Additional concentration through immunoprecipitation was performed on the cell lysate harvested as described above to enhance the detection of any small Flag-tagged peptides. In short, 4 μg of antibodies against the Flag tag, i.e., ANTI-FLAG® M2, Clone M2 (Sigma), were bound to protein G on the beads. The beads were combined with the cell lysate as described above or with 1 mL of supernatant. The incubation time was extended to 1.5 h. Immunoprecipitated products were eluted from the beads by incubation at 70°C for 10 min in 20 μL of Elution buffer from the kit, combined with 7 μL of 4x NuPAGE® LDS Sample Buffer and 3 μL of 10x NuPAGE® Sample Reducing Agent (Invitrogen).

All samples were applied to separate wells in a NuPAGE® Novex™ 12% Bis-Tris Protein Gel (Invitrogen). MagicMark™ XP Western Protein Standard and/or SeeBlue™ Plus2 Pre-stained Protein Standard (both Invitrogen) were included in parallel wells. Electrophoresis was run in XT-MOPS running buffer (BioRad) in an XCell SureLock® Mini-Cell (Invitrogen) at 200 V for 70 min. Subsequently, the proteins were transferred to a PVDF membrane (BioRad) using a semi-dry blotter at 20 V for 20 min. Proteins were detected by immunostaining using anti-TurboGFP(d) antibody (Evrogen) and ANTI-FLAG® M2, Clone M2 (Sigma), both at a working solution of 1 μg/mL, in combination with ECL™ anti-rabbit or -mouse IgG HRP-linked (Amersham), diluted 1:4000 or 1:1000, respectively, and Opti-4CN substrate (BioRad).

### 2.11 LC–MS and data analysis

p33 and its smaller peptide products detected in Western blots were analyzed by LC–MS. Protein products were harvested from cells and supernatants and prepared as described above, including the additional immunoprecipitation step to analyze proteins from supernatants. The samples were applied to separate wells in a 4–20% Criterion™ TGX™ Precast Midi Protein Gel for analyses focusing on N-terminal peptides and a 4–12% Criterion XT Bis-Tris protein gel for C-terminal peptides (BioRad). Electrophoresis was run in XT-MOPS running buffer (BioRad) in a Criterion™ Vertical Electrophoresis Cell (BioRad) at 200 V for 55 min. The gel was subsequently stained overnight with Coomassie using the Pierce™ Mini Gel Power Staining Kit (Thermo Scientific), with three repeated incubations for 40 min for destaining, all at 4°C with gentle shaking. Target bands representing full-length p33 and shorter products were excised with a scalpel, using a Western blot of a duplicate SDS-PAGE gel as a guide. For in-gel tryptic digestion, the gel pieces were digested with 0.2 μg trypsin GOLD (Promega) in 80 μL of 25 mM ammonium bicarbonate pH 7.8 for 16 h at 37°C. The digestion was stopped by adding 5 μL of 50% formic acid, and the generated peptides were purified using a 10-μl OMIX C18 micro-SPE pipette tip (Agilent) and dried using a Speed Vac concentrator (Concentrator Plus, Eppendorf). The samples were analyzed by LC–MS using a timsTOF Pro (Bruker Daltonik), which was coupled online to a nanoElute nanoflow liquid chromatography system (Bruker Daltonik) via a CaptiveSpray nanoelectrospray ion source. The dried peptides were dissolved in 4 μL of 0.1% formic acid, and 2 μL of the sample was injected. The peptides were separated on a reversed-phase C18 column [15 cm × 75 μm, 1.7 μm, Aurora Elite (IonOpticks)]. Mobile phase A contained water with 0.1% formic acid, and acetonitrile with 0.1% formic acid was used as mobile phase B. The peptides were separated by a gradient from 0 to 35% mobile phase B over 30 min at a flow rate of 200 nL/min at a column temperature of 50°C. MS acquisition was performed in DDA-PASEF mode. The capillary voltage was set to 1.5 kV with a mass range of 100 to 1700 m/z. The number of PASEF ranges was set to 20, with a total cycle time of 1.16 s, charge up to 5, target intensity of 20,000, intensity threshold of 1,750, and active exclusion with release after 0.4 min. An inversed reduced TIMS mobility (1/*k*_0_) of 0.85–1.40 *Vs*/cm^2^ was used with a range time of 100 ms, an accumulation time of 100 ms, a duty cycle of 100%, and a ramp rate of 9.51 Hz. Precursors for data-dependent acquisition were fragmented with an ion mobility-dependent collision energy, which was linearly increased from 20 to 59 eV.

The LC–MS data were searched against the piscine myocarditis virus UniProt database (142 entries) using Mascot 2.7.0.1. The following parameters were used: digestion enzyme, trypsin; maximum missed cleavage, 1; fragment ion mass error tolerance, 0.03 Da; parent ion error tolerance, 15 ppm. Oxidation of methionine, propionamide formation of cysteines, and acetylation of the N-terminus were specified as variable modifications. Scaffold 5.1.2 (Proteome Software Inc.) was used to validate MS/MS-based peptide and protein identifications. Peptide identifications were accepted if established with greater than 95.0% probability by the Scaffold Local FDR algorithm. Protein identifications were accepted if established with a false-discovery rate better than 1% and containing at least 2 identified peptides.

### 2.12 Inhibition of ER–Golgi traffic using Brefeldin A

EPC cells were transfected by electroporation in 100-μl tips as described above, and the cells from two electroporations were combined in one well of a six-well multidish. Brefeldin A (Sigma Aldrich) was added to a final concentration of 1 μg/mL at 3 hpt. At 3 dpt, cell culture medium supernatant and cells were harvested. The medium was centrifuged at 13,000 rpm in a microcentrifuge at 4°C for 15 min to remove cell debris. The cells were harvested in 200 μL CelLytic M (Sigma) per well. Cell harvest and analyses of proteins in both medium and cells by immunoprecipitation (medium only) and Western blot were then performed as described above.

### 2.13 Statistical analyses

The LDH cell death curves for the different p33 variants were compared using linear regression with LDH as the dependent variable and time (hours post transfection) and different p33 variants as independent variables (with *p*-value < 0.05 considered significant). Analysis was performed using GraphPad Software Inc. version 7.0b.

### 2.14 Data availability

The sequences of ORF3 from the 11 wild-type p33s (wt1-11) are published in GenBank under accession numbers OQ615313–OQ615323.

## 3 Results

### 3.1 p33 expression is cytotoxic to fish cells

**Figure 1:**
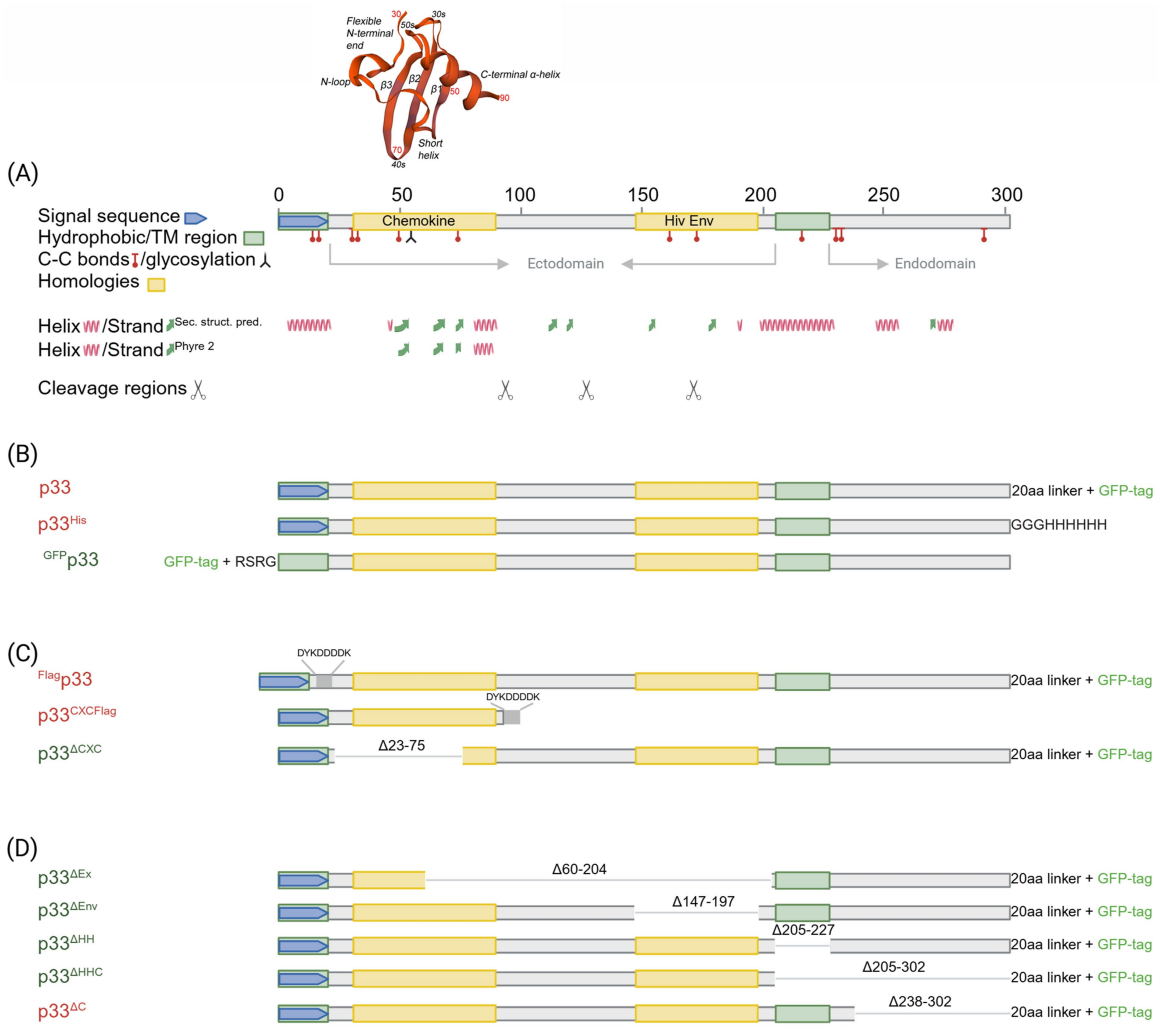
*In silico* predicted characteristics of PMCV p33 and schematic overview of sequence characteristics of various p33 recombinant mutants used in sections 3.1–3.6. **(A)** Schematic overview of the positions of features and characteristics found in the p33 sequence using *in silico* predictions. Signal sequence, chemokine-like domain, regions of high hydrophobicity/putative transmembrane (TM) domain, and secondary structure (*α*-helix, *β*-strand) prediction as given by Sandlund et al., 2021. Cysteines present in all parts of the p33 amino acid sequence, representing sites predicted for putative disulfide bonds and a predicted glycosylation site, are indicated. A region with weak homology to part of the HIV-1 Env protein, resulting from homology searches under low stringency, is also shown. Hypothesized regions for cleavage sites predicted by the size of peptide products Cp1-3 vs. amino acid sequence length are indicated by scissor symbols. A 3D model is included for the chemokine-like domain, predicted against CXC chemokine 3, and shows characteristic secondary structures described as a flexible N-terminal end including the two CXC motif cysteines and an N-loop, followed by a short helix, three antiparallel β-strands (β1–β3), and finally one α-helix in the C-terminal end. Residue numbers relating to full-length p33 are given in red for every twentieth residue in the model. **(B)** Overview of p33, including various uses of tags and tag-linkers used in sections 3.1, 3.2, and 3.3. The 20aa linker for the C-terminal GFP tag of p33 and mutants given in (C)-(D) includes the amino acids LKLRILQSTVPRARDPPVAT. The linker for the C-terminal 6x His tag of p33^His^ and the N-terminal GFP tag of ^GFP^p33 are as given in the figure. **(C)** Overview of p33 recombinant mutants used to study the N-terminal end of p33 in sections 3.4 and 3.6, i.e., the chemokine-like domain, including a full-length p33 with an N-terminal Flag and the standard C-terminal GFP (^Flag^p33), and p33 recombinant mutants expressing the separated chemokine-like domain with a C-terminal Flag tag (p33^CXCFlag^) and a variant with a deletion of amino acids important for the structure of the chemokine-like domain (p33^ΔCXC^). Specific numbers of deleted residues are given. **(D)** Overview of p33 recombinant mutants with various deletions used to study the C-terminal end of p33 in section 3.5. p33^ΔEx^ – deletion of the majority of the region predicted as extracellular in a putative transmembrane protein; p33^ΔEnv^ – deletion of amino acids with weak homology to the HIV-1 Env protein. p33^ΔHH^ – deletion of the region of high hydrophobicity; p33^ΔHHC^ – deletion of the region of high hydrophobicity and the following C-terminal end; p33^ΔC^ – deletion of the C-terminal end after the region of high hydrophobicity. Specific numbers of deleted residues are given. All p33 variants used in analyses include a C-terminal GFP tag unless otherwise specified by variant naming or in the text, i.e., all p33 variants with no indication of a tag in naming (p33, p33^ΔCXC^, etc.) include the C′ GFP tag as shown. The naming is provided with color coding, where red indicates a resulting cytotoxic phenotype and green indicates low or no resulting cytotoxicity (see text for details). Created in https://BioRender.com.

As there is a general lack of cultured cells able to propagate PMCV with efficiency suitable for experimental trials, we studied the functional characteristics of the p33 protein *in vitro* through recombinant expression from a plasmid vector. Although attempts have been made to generate antibodies specific to p33 through several procedures, there are currently no efficient and specific antibodies to p33 available. Hence, the majority of experiments were performed using p33 with a C-terminal green fluorescent protein (GFP) tag for real-time microscope observations of the protein expression, unless otherwise stated. An N-terminal GFP tag, His tag, and/or Flag tag were included as appropriate for controls or double-tagging. To understand the various *in silico* predicted features and characteristics found in the p33 sequence, specific parts of p33 were included in various construct expressions. All p33 constructs used in the study are shown in Figure 1, including a schematic overview of *in silico* predicted features and characteristics found in the p33 sequence. The fish cell lines, CHH-1, EPC, and CHSE, were transfected to express the p33 variants.

**Figure 2:**
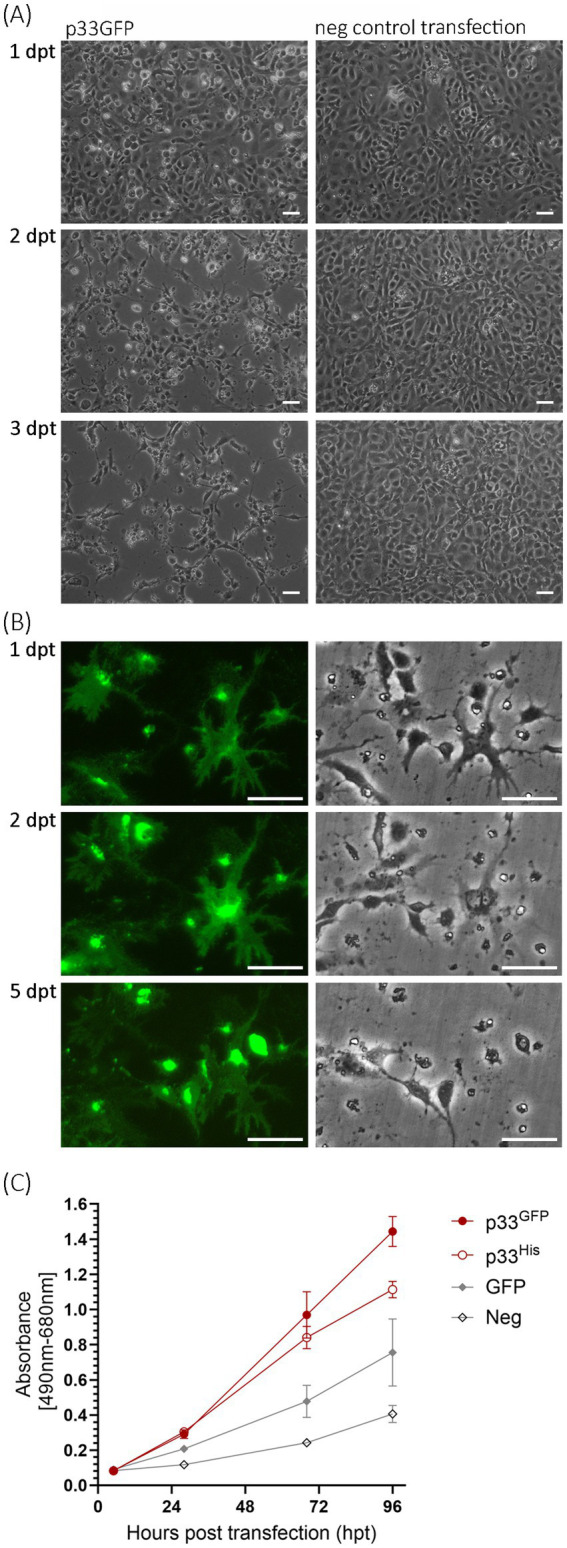
Microscopy studies of EPC cells transfected to express p33. **(A)** Phase contrast imaging of the cell monolayer at 1, 2, and 3 days post-transfection (dpt) showing the progressive cytopathic effect of transfected cells (left) compared to negative control cells (mock transfected with no plasmid addition; right). Scale bar = 50 μm. **(B)** Imaging of single cells expressing p33 by fluorescence (left) and phase contrast (right) microscopy at 1, 2, and 5 dpt. Early time points show that protein expression morphology coincides with the morphology of the cells, but over time the cells disappear and leave the protein behind as an “imprint” of the cell. Neighboring cells exhibit increasing fluorescence from the expressed protein with the C-terminal GFP tag, but this also diminishes with time. Corresponding full-size images are included in Supplementary Figure S1, along with images of parallel control cell cultures expressing PMCV capsid similarly with GFP tag and negative control cells. Scale bar = 50 μm. **(C)** Lactate dehydrogenase (LDH) assay measuring the release of the cytosolic enzyme LDH into the surrounding cell culture media as an indicator of plasma membrane damage, for p33 with either GFP or His C-terminal tags, compared to cells expressing GFP only or negative cell transfection control. Absorbance increases with time at a significantly (*p* < 0.001) higher rate in p33-expressing cells compared to controls. There is no significant difference (*p* > 0.05) between p33 with either GFP or His tags up to 68 h post-transfection (hpt), although at 96 hpt, LDH levels are significantly higher in the GFP tag than in the His tag (*p* < 0.05). Representative images from microscopy of the cell cultures with phase contrast indicating the degree of CPE and parallel fluorescence microscopy showing protein expression from the GFP tag may be found in Supplementary Figure S2A.

We began our studies by performing a time-course experiment focusing on changes in cellular morphology following the expression of p33 in cell culture over 1–3 days post-transfection (dpt). We used EPC cells, with imaging conducted using phase contrast and fluorescent microscopy in parallel. We observed that the transfected cells appeared rounded and detached from the substratum, with a halo visible as early as 1 dpt by phase contrast microscopy (Figure 2A). At 2 dpt, cellular debris was apparent, and spindle-shaped cells and areas devoid of cells became evident. By 3 dpt, the number of attached cells continued to decrease, and the remaining cells were spindle-shaped (Figure 2A). These phenotypic changes resembled a general cytopathic effect (CPE) of virus-induced changes observed with many viruses. In a second assay, we imaged specific single cells in a small fixed area over 5 dpt, showing that fluorescence intensity increased over time, corresponding with a gradual loss of cellular integrity and details during the first 2 dpt (Figure 2B; corresponding full-size images are included in Supplementary Figure S1). The green fluorescence from the protein outlined the cell body and perimeter, and when the cell body was undetectable by phase contrast microscopy, an “imprint” of the cell was visible by fluorescent microscopy (Figures 2B, 5 dpt).

Next, we assessed the kinetics of cellular integrity loss by measuring the release of lactate dehydrogenase (LDH) from the cells over a 96 h period post-transfection. LDH was measured in the cell culture supernatant. Both p33 with a C-terminal GFP tag (p33) and His tag (p33^His^, Figure 1B) were included in the analyses and showed a significant increase in cellular LDH release compared to controls (Figure 2C), supporting the observed increase in the CPE-like phenotype (Figures 2A,B).

**Figure 3:**
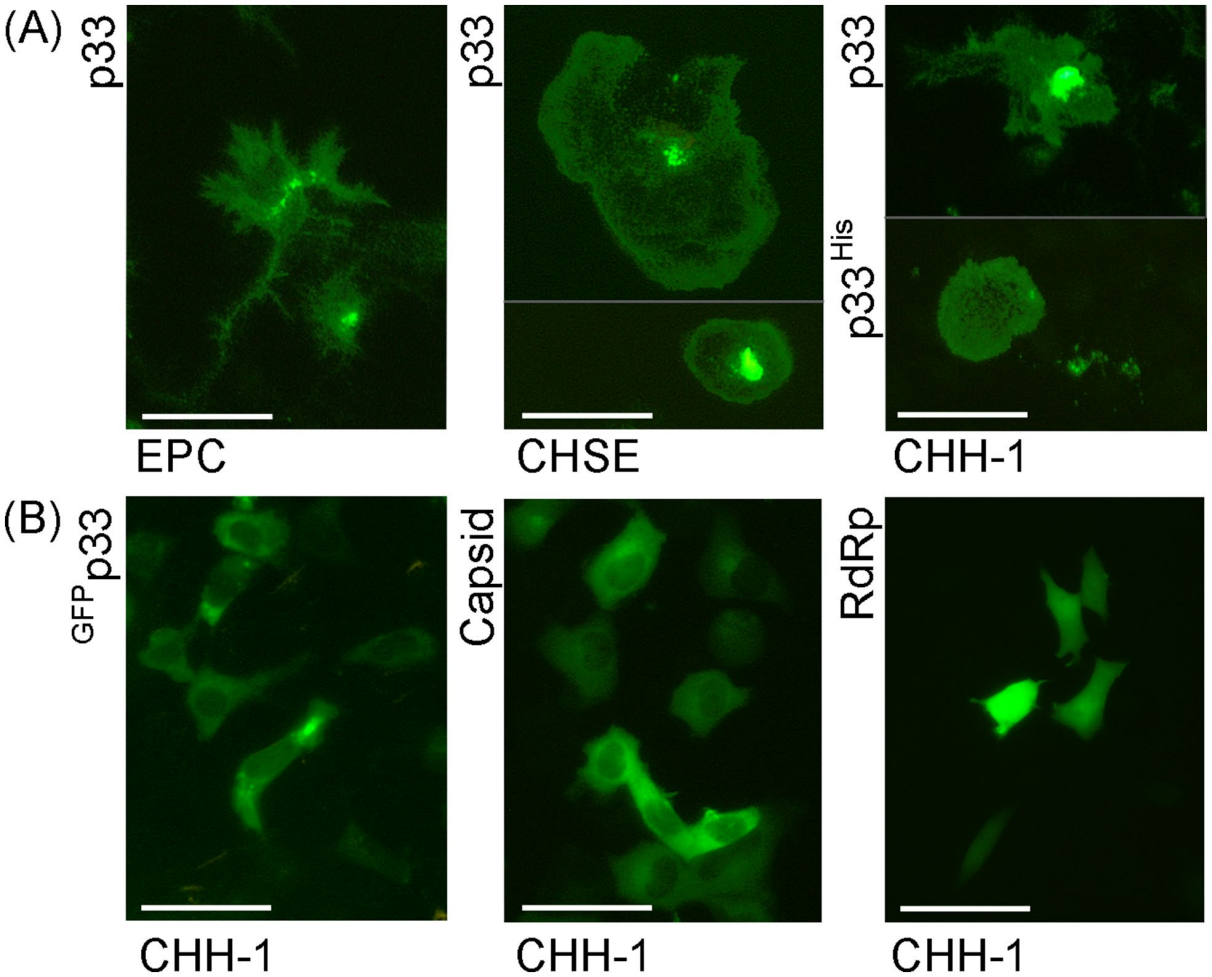
Examples of the characteristic appearance of fluorescence from PMCV p33 protein with a C-terminal GFP tag (p33) in piscine cell lines. **(A)** p33 expression in EPC, CHSE, and CHH-1. For CHSE, an example with extreme size is included, together with one of typical size. For p33 expression in CHH-1, an example of p33 with a C-terminal His tag (p33^His^) visualized using IFAT with anti-His antibodies is included in addition to the p33 with the standard C-terminal GFP tag. A parallel image of the anti-His IFAT stained CHH-1 cells expressing p33^His^, including Hoechst-stained nuclei of all cells is included in Supplementary Figure S3 for confirmation of the specificity of the antibodies. **(B)** CHH-1 expressing p33 with an N-terminal GFP tag (^GFP^p33) or PMCV capsid and RdRp, both with a C-terminal GFP tag for comparative controls. Images were captured at varying time points (1–4 dpt) but reflect characteristic examples at any time point. Scale bar = 50 μm.

The green fluorescence of the expressed protein exhibited high variability in its distribution, intensity, and morphological appearance among cells post-transfection (Figure 3A), compared to control cells expressing PMCV capsid or RdRp under identical conditions (Figure 3B). Although the phenotypic effect on the p33-expressing cells was overall comparable, independent of the cell line, the fluorescence appearance of the expressed protein showed slightly different characteristics depending on the fish cell line used (Figure 3A). The most common characteristics of the protein expression included changes in cell morphology and varying increases in cell size and the size of the apparent protein structure area, as visualized by fluorescence from the GFP tag. For CHH-1, this resulted in a mean size 1.6–1.9 times larger than control cells (measurement details and example images may be found in Supplementary Data Sheet S1), and the appearance was also characterized by irregular protrusions or extensions from the cell periphery. For EPC cells, the end phase usually resulted in an imprint of the irregular cell shape. In contrast, for CHH-1 and CHSE, larger structures were occasionally observed, indicating a three-dimensional hemispherical shape (Figure 3A). To confirm that the characteristic expression was specific to p33, controls included not only the expression of Capsid and RdRp but also a construct in which the C-terminal GFP tag was replaced with a short His tag (p33^His^, Figure 3A), as well as a switch of the tag from the C-terminal to the N-terminal position (Figure 3B, ^GFP^p33). Only the switch of the GFP tag to the N-terminal position affected the expression characteristics. ^GFP^p33 indicates that the position of the tag impacts the distinctive expression morphology seen with the C-terminal tag, resulting in diffuse fluorescent cytoplasm outlining the normal cell shape. This may be explained by the N-terminal GFP tag disturbing the function of the putative signal sequence found at the N-terminal end, thereby affecting correct expression. Finally, comparison of the appearance of expressed p33 in (Figure 3A) to cells expressing PMCV capsid and RdRp with a similar C-terminal GFP tag confirms that the distinct expression morphology is specific to p33, as the resulting fluorescence from the expressed control proteins is restricted within the perimeter of the cell, including the nucleus for the RdRp (Figure 3B).

**Figure 4:**
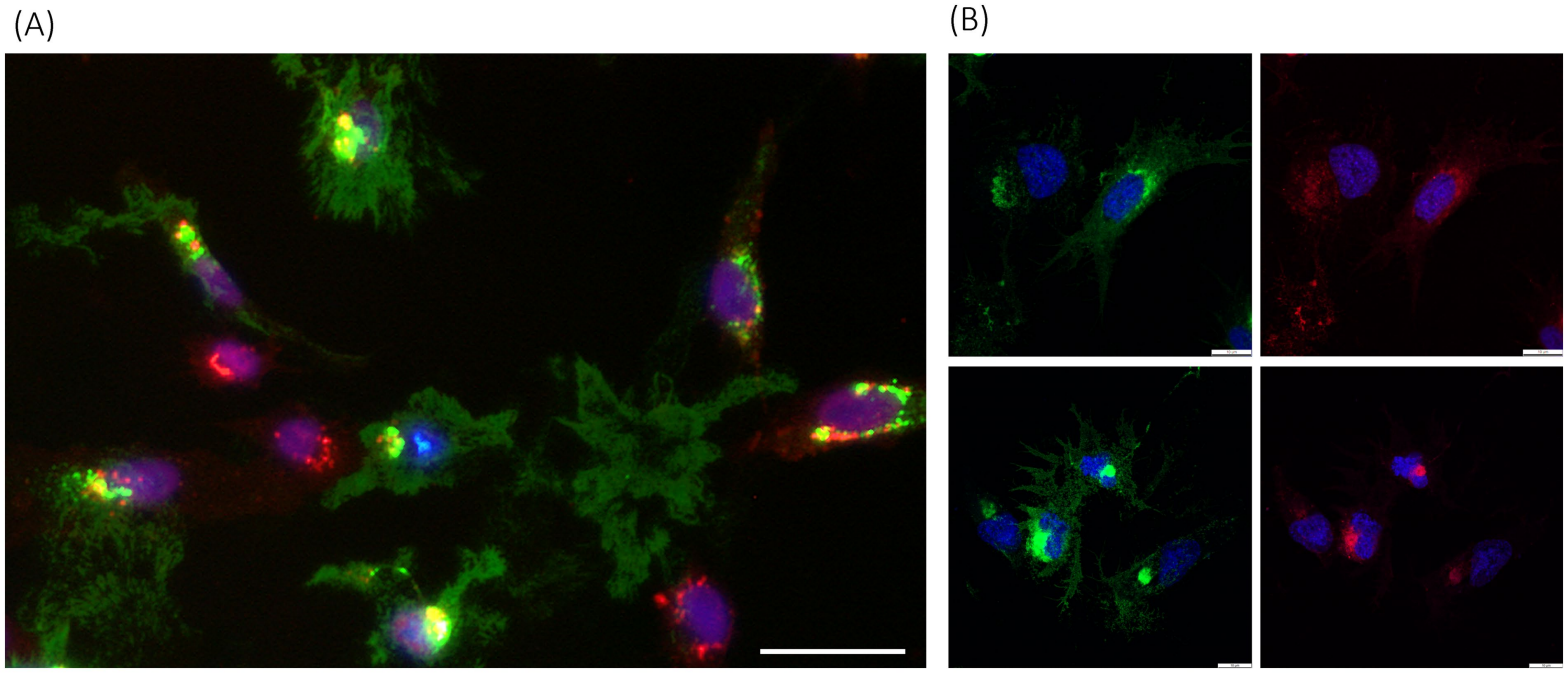
PMCV p33 expression and correlation with cell membranes, including Golgi. **(A)** p33 expressed with a C-terminal green-fluorescent tag in CHH-1 cells (3 dpt), imaged using standard inverted fluorescence microscopy, with red fluorescent WGA-stained cell membranes, including membranes of the Golgi, and blue fluorescent Hoechst-stained nuclei. Yellow fluorescence indicates co-localization of the p33 and Golgi. Examples include cells with a low degree of protein expression only in dense perinuclear granules, seen as spots with green staining of high fluorescent intensity located around the nuclei, correlating with Golgi, but also examples of dense accumulation of p33 close to the nucleus, correlating with Golgi. In addition, examples of p33 expression with only remnants of cell debris scattered in between, are also shown. Cells with no expression of p33 are found with red fluorescent Golgi and blue fluorescent nuclei only. Scale bar is 50 μm. **(B)** Examples of p33 expression in the EPC cell line (3 dpt) imaged using high-resolution confocal microscopy at low protein expression levels (upper panel) and high expression levels (lower panel). Parallel images show green fluorescence from p33 C-terminal GFP tag (left images) and red fluorescence indicates cell membranes, including high specificity for membranes of the Golgi with higher intensity close to the nucleus (right images), both combined with blue staining of nuclei. Scale bar is 10 μm.

Also, a general characteristic of p33 expression was the accumulation of dense fluorescence from the protein, in addition to the appearance of cell morphology and structures. The cells were stained with fluorescent wheat germ agglutinin (WGA), which binds specifically to sialic acid and N-acetylglucosaminyl residues on the plasma membrane and trans-Golgi network, to better characterize the localization (Figure 4). In cells with intact boundaries, co-localization of p33 with WGA-stained perinuclear structures, expected to be consistent with the Golgi, was observed. A few cells also exhibited lower protein expression efficiency, characterized by dense, perinuclear green fluorescent granules of varying size and intensity. At the same time, cellular integrity was maintained (Figure 4A). Additionally, this fluorescent p33 co-localized with WGA-stained Golgi.

### 3.2 Cytotoxicity exhibits features of both apoptotic and necrotic character

**Figure 5:**
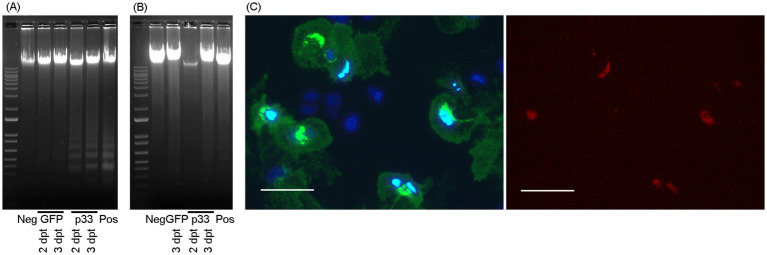
Cytotoxic cell death mechanisms. DNA fragmentation in EPC **(A)** and CHH-1 **(B)** cells at 2 and 3 dpt for the expression of p33 (with GFP tag) visualized through gel electrophoresis separation. Controls include staurosporine-treated cells (positive control), cells transfected to express GFP only, and cells mock transfected with no plasmid (negative control). Marker – 1 kb+ (Invitrogen). **(C)** CHH-1 cells similarly transfected to express p33 were also subjected to double staining using Hoechst and propidium iodide at 2 dpt. Left image: Hoechst-stained nuclei. Normal nuclei are shown in dark blue, while condensed or degenerated nuclei of dead cells are shown in intense light blue. Right image: Propidium iodide stain selective for the nuclei of cells with compromised membranes (i.e., necrotic cells) corresponds to the condensed/degenerated intense light blue nuclei shown in the left image. Scale bar 50 μm.

The observed LDH leakage post-transfection indicated compromised cellular membranes following p33 expression. Since the LDH assay provides limited information on the underlying mechanisms causing cell damage or death, we proceeded with studies of DNA fragmentation, a hallmark of apoptosis. We included EPC and CHH-1 cells expressing p33 (with GFP tag) and sampled at 2 and 3 dpt. Controls included cells expressing GFP only, in addition to mock-transfected cells with no plasmid (negative control) and staurosporine-treated cells (positive control). The p33-expressing EPC cells showed DNA fragmentation comparable to the staurosporine-treated positive control cells, while EPC cells expressing GFP showed only a weak indication of fragmentation (Figure 5A). For CHH-1 cells, staurosporine treatment resulted in morphological changes similar to those observed in EPC cells. However, no clearly apparent DNA fragmentation was observed in this control (Figure 5B). Similarly, CHH-1 cells expressing p33 or GFP did not show apparent DNA fragmentation (Figure 5B). For the p33 expression in CHH-1 cells, we then included double staining with Hoechst and propidium iodide (PI) and examined the effect over time at 1, 2, 3, and 6 dpt. Hoechst dye stains the nuclei of viable and dead cells, while only the nuclei of cells with compromised or perforated membranes are stained with PI, indicating necrotic and/or late-stage apoptotic cells. At 1 and 2 dpt, CHH-1 cells expressing low levels of p33 showed normal nuclei and were positive for Hoechst staining only. In contrast, cells with intense p33 staining were positive for both Hoechst and PI, displaying condensed and/or fragmented nuclei (examples from 2 dpt are shown in Figure 5C; overview scaled images of all time points are included in Supplementary Figure S4). From this, we interpret that p33 may induce a phase of apoptotic events in the cells, possibly followed by post-apoptotic or necrotic phases early after p33 expression. However, this may vary depending on the cell type.

### 3.3 p33 is processed into sub-peptides

**Figure 6:**
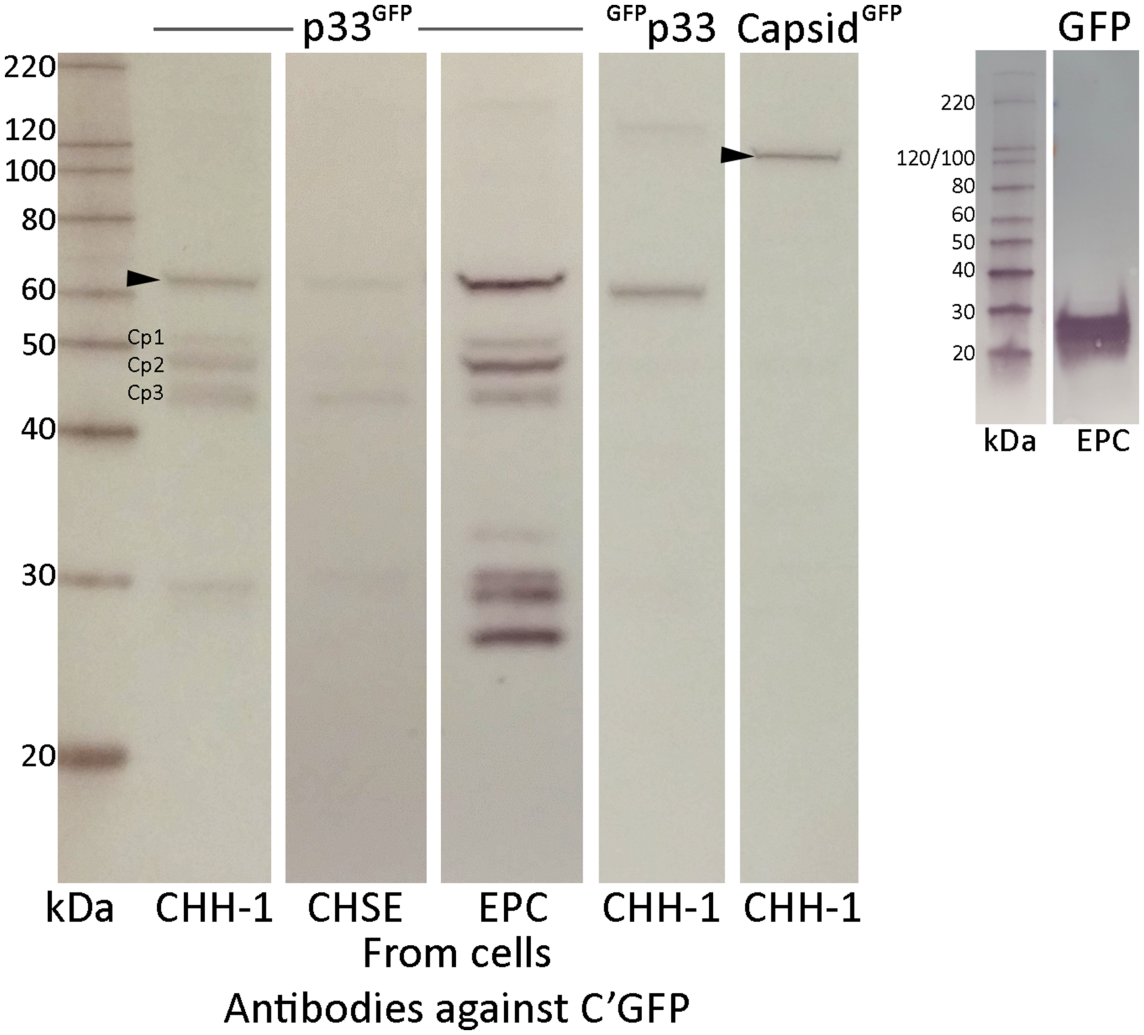
Western blot studies of protein harvested from various cell lines after recombinant expression of p33 with a C-terminal GFP tag, showing full-length protein and shorter p33 peptide products. Cell lines are transfected using the same conditions and show varying concentrations of expressed full-length protein p33 and the smaller peptide products. Main peptide products are named Cp1-3 due to their representation as peptide products from the C-terminal end of the protein. p33 with an N-terminal GFP tag and PMCV capsid with a C-terminal tag are included as controls. Proteins are detected using antibodies against the GFP tag. The size of full-length protein products for both p33 and capsid, corrected for the combined GFP tag, is indicated by an arrowhead (61 and 120 kDa, respectively). Full-length N-terminal tagged p33 is correctly sized slightly smaller than p33 with a C-terminal GFP due to a shorter amino acid link used when positioning the tag at the N-terminal end of the ORF3 protein. A separate run of a Western blot of protein harvested from EPC cells after recombinant expression of GFP only is included for comparison. MagicMark™ XP Western Protein Standard is used for size comparison in kilodaltons (kDa).

To further characterize the p33 expressed in cells, we examined lysates from transfected cells by Western blot, using an anti-GFP antibody for detection. Lysates from PMCV capsid-expressing cells were included for comparison. We found that the p33 protein was expressed at the expected size (61 kDa corrected for the included tag) (Figure 6), but several smaller products were detected in addition to the 61 kDa full-length product. Three smaller protein bands of approximately 52, 48, and 43 kDa, along with one at approximately 29 kDa, were observed in all the cell lines tested. EPC had higher transfection efficiency, resulting in a greater concentration of proteins, allowing us to detect additional smaller products below 29 kDa (Figure 6). However, p33 with the N-terminal GFP tag (^GFP^p33) did not show observable protein bands below the full-length product (Figure 6). Interestingly, this N-terminal GFP-tagged p33 also lacked the cytotoxic phenotype (Figure 3).

Since the full-length p33 and the smaller products were detected using antibodies against the GFP tag fused to the C-terminal end of p33, the smaller-sized bands likely represent products with N-terminal portions removed from the full-length protein. The resulting 52, 48, and 43 kDa C-terminal peptides are referred to as Cp1, Cp2, and Cp3, respectively, hereafter. The products at sizes below 30 kDa may represent either the GFP tag alone (26 kDa) or those including the C-terminal linker (28 kDa). From these findings, we have a strong indication that p33 was cleaved into shorter fragments, and cleavage was potentially linked to cytotoxicity.

### 3.4 p33-derived chemokine-like peptides are essential for cytotoxicity and are secreted from the cells

The *in silico* analyses showed that a CXC chemokine-like domain follows the signal sequence at the N-terminal end of p33, and this domain, along with the subsequent part of the protein sequence leading up to the high hydrophobicity/transmembrane domain, is predicted to be extracellular (Figure 1A; Sandlund et al., 2021). In tissue cells, chemokines are produced as small (8–12 kDa) secretory molecules with an N-terminal signal sequence that is removed once the chemokine is synthesized in the endoplasmic reticulum (ER). From the above, it was indicated that N-terminal localization of the GFP tag (^GFP^p33) affected the expression characteristics of p33 as ^GFP^p33 lacked the cytotoxic phenotype (Figure 3). Additionally, this variant did not show observable protein bands below the full-length product (Figure 6), indicating that proteolytic cleavage was affected, which may be explained by the N-terminal GFP tag influencing the function of the N-terminal signal sequence.

To explore whether the predicted p33-derived chemokine-like domain is secreted, we expressed full-length p33 with a C-terminal GFP tag in EPC. In the construct used, we included a Flag tag at the N-terminal end of the chemokine-like domain immediately following the signal sequence (^Flag^p33; see schematic overview of p33 constructs, Figure 1C). Expression of this double-tagged ^Flag^P33 variant was confirmed by the observation of green fluorescence from the GFP tag (not shown). The expression characteristics were indistinguishable from those of standard GFP-tagged p33 without the Flag tag (p33) (shown previously in Figures 2–5).

**Figure 7:**
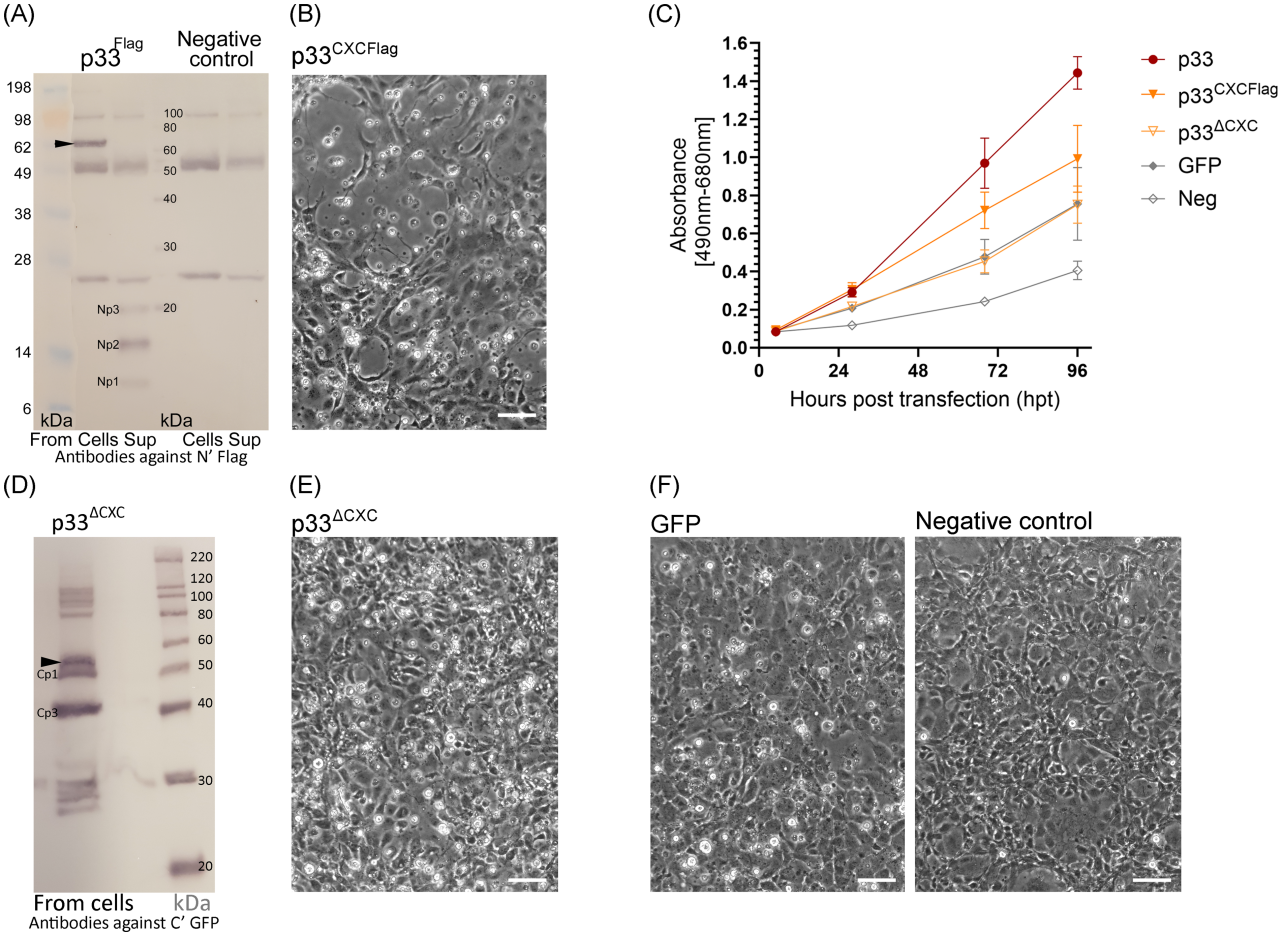
Characteristics of chemokine-like domain p33 peptide products. **(A)** Western blot studies of protein and peptide products harvested from cells and extracellular supernatants from p33-expressing cells and negative control cells. Proteins are detected using antibodies against the Flag tag inserted immediately after the signal sequence, preceding the chemokine-like domain. The full-length p33 protein product is indicated by an arrowhead (including the signal sequence and N′ Flag and C′ GFP tag, 62 kDa, respectively) with presence in cells. Peptide products are named Np1-3 due to representing peptide products from the N-terminal end of the protein and are present in the supernatant. SeeBlue™ Plus2 and MagicMark™ XP Western Protein Standards are used for size comparison in kilodaltons (kDa). Unspecific protein products present in all lanes are due to contamination with heavy (approximately 50 kDa) and light chains (approximately 25 kDa) from antibodies used in immunoprecipitation procedures to concentrate the specific products for detection. **(B)** Phase contrast imaging of the cell monolayer of EPC cells transfected to express p33^CXCFlag^ at 4 dpt, showing CPE of cells expressing p33^CXCFlag^ [see **(F)** for control cells]. **(C)** Lactate dehydrogenase (LDH) assay measuring the release of the cytosolic enzyme LDH into the surrounding cell culture media as an indicator of damage to the plasma membrane for p33^CXCFlag^ and p33^ΔCXC^ compared to p33 (all with tags as described in the text) and also to cells expressing GFP only or negative cell transfection control (all as described previously in Figure 2C). Absorbance from cells expressing p33^CXCFlag^ increases significantly with time, but at a slower rate than for p33-expressing cells (*p* < 0.001). The absorbance from p33^ΔCXC^-expressing cells increases significantly over time (*p* < 0.001) but remains at indiscernible levels compared to GFP control cells. Representative images from microscopy of the cell cultures with phase contrast indicating the degree of CPE and parallel fluorescence microscopy showing protein expression from the GFP tag may be found in Supplementary Figure S2B. **(D)** Western blot study of protein harvested from cells expressing p33^ΔCXC^ with detection using antibodies against the GFP tag. The size of the full-size protein product is indicated by an arrowhead for full-length p33^ΔCXC^ (corrected for deletion in the mutant), and smaller products correlating with Cp1 and Cp3 are indicated. MagicMark™ XP Western Protein Standards are used for size comparison in kilodaltons (kDa). **(E)** Phase contrast imaging of the cell monolayer of EPC cells transfected to express p33^ΔCXC^ at 4 dpt, showing no clear CPE compared to p33^CXCFlag^ in **(B)** and which have replicated to confluency similar to the controls shown in **(F)**. **(F)** Control cells: GFP-expressing cells and negative (mock transfected with no plasmid addition) cells. All scale bars are 50 μm.

The cell cultures expressing p33 were studied for the presence of any N-terminal Flag-tagged peptides, both intra- and extracellularly, using Western blot analyses of cell lysates and cell culture supernatants (Figure 7A). A protein consistent with the size of full-length Flag and GFP-tagged p33 was detected in the cells. Extracellularly, three short peptides of approximately 9–20 kDa were detected, named Np1-3 (N-terminal peptides), from the smallest to the largest, respectively. Due to the resolution of the size of protein bands in the molecular weight standard used for size comparison, the exact size prediction of these peptides and the sizes previously described for their Cp1-3 counterparts (Figure 6) is inaccurate. However, it is interesting to note that the size of the largest of the C-terminal peptides (Cp1, approximately 52 kDa) and the smallest N-terminal peptide (Np1, approximately 9 kDa) together roughly constitute the size of a full-length protein (61 kDa). A similar observation is seen for Cp2 and Cp3 combined with Np2 and Np3, respectively.

To further investigate the role of the CXC chemokine-like domain, we expressed the chemokine-like region separately. This construct included the N-terminal signal sequence and the chemokine-like domain, now with a Flag tag added at the C-terminal end following the predicted *α*-helix (Figure 1C, p33^CXCFlag^). Expression and secretion of p33^CXCFlag^ were confirmed by detection of a protein product consistent with the expected molecular weight by Western blot from the supernatant shown later in Section 3.7, Figure 10A, p33^CXCFlag^ under conditions without Brefeldin A (BFA-). Interestingly, expression of p33^CXCFlag^ resulted in a cytotoxic phenotype, as observed by phase contrast microscopy (Figures 7B,F). This was supported by a significant increase in LDH release over time, although at a slower rate than for the full-length p33 (Figure 7C). A p33 mutant with significant portions of the chemokine-like region deleted (p33^ΔCXC^), still including the C-terminal GFP tag, was constructed for comparison. By Western blot, the full-length product of p33^ΔCXC^ was detected, along with additional smaller products corresponding to Cp1 and Cp3, and possibly separated GFP products below 30 kDa (Figure 7D), as seen earlier (Figure 6). However, no Cp2 was detected. Studies of cell culture expression supported a role for this domain in cytotoxicity, as this variant lacking the chemokine-like domain did not induce a cytotoxic phenotype (Figures 7E,F). Furthermore, LDH release was at levels comparable to those found for recombinant protein expression controls (i.e., GFP-expressing cells) (Figure 7C).

### 3.5 The smaller peptide products Cp1-3 and Np1-3 are specific to p33

**Figure 8:**
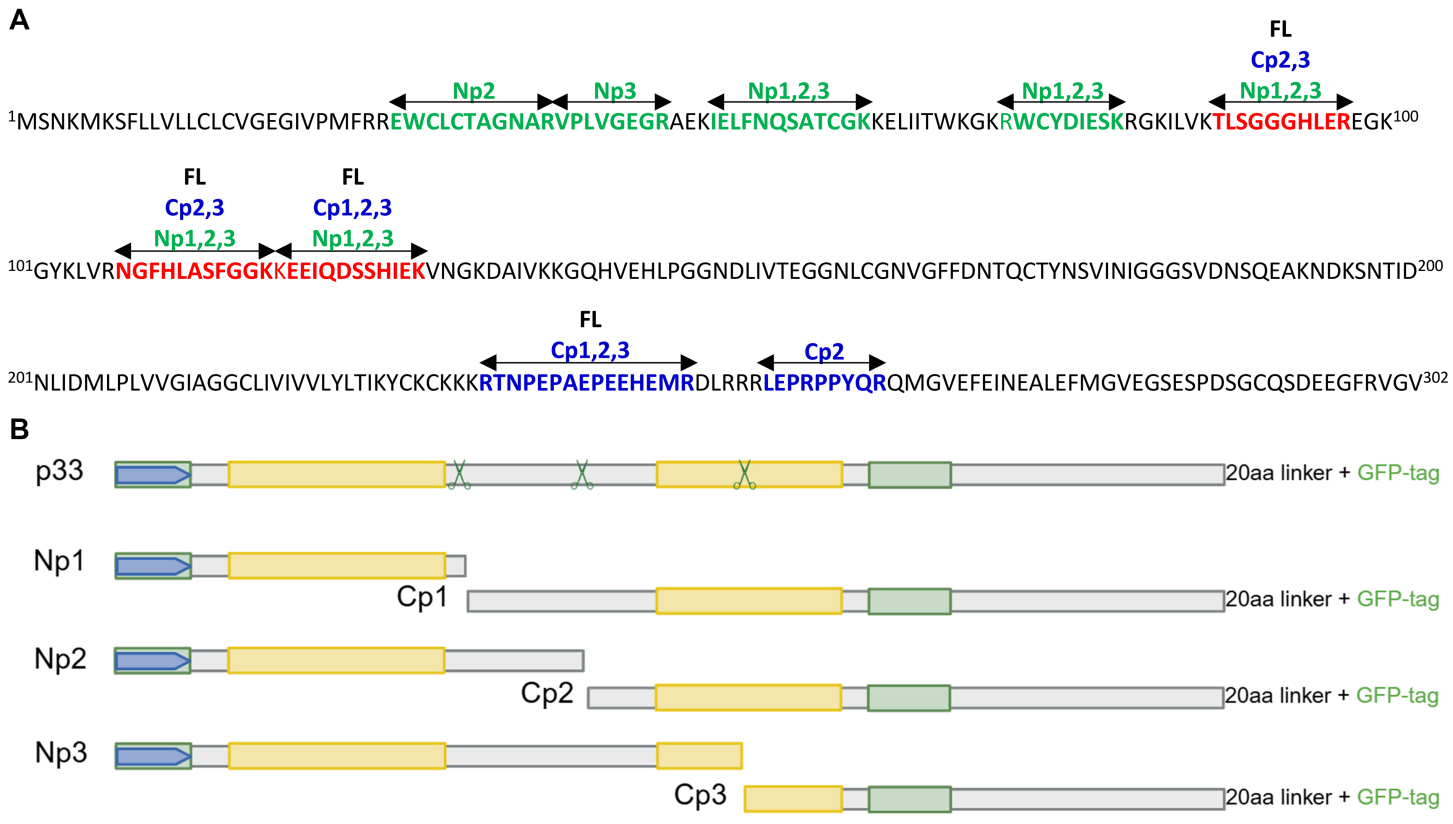
**(A)** LC–MS analyses of products from EPC cells expressing p33 with both N-terminal Flag and C-terminal GFP tags. p33 peptide sequences were identified by separation on SDS-PAGE from cell culture supernatant (Np1-3) and cells (full-length p33 and Cp1-3), subjected to in-gel tryptic digestion and MS/MS identification as shown. Sizes of 61 (full-length, FL), 52 (Cp1), 48 (Cp2), 43 (Cp3), 20 (Np3), 15 (Np2), and 9 (Np1) kDa are shown in different colors (sequences in green for Np, in blue for Cp, and red for Np and Cp). See Figures 6, 7A for visualization of band sizes excised. **(B)** Schematic overview of full-length p33 and sub-peptides Np1-3 and corresponding Cp1-3. Hypothesized regions for cleavage sites in p33, predicted by the size of peptide products vs. amino acid sequence length, are indicated by scissor symbols. For other schematic indications of protein sequence domains, see Figure 1A. Created in https://BioRender.com.

The product bands expected to be full-length p33 and its Cp1-3 and Np1-3 peptide products were assessed for specificity to p33 by separation on SDS-PAGE, in-gel tryptic digestion of excised bands representing all the products, and subsequent LC–MS analyses. p33 peptide sequences were identified by MS/MS from all bands (Figure 8A).

To narrow down the most likely region of p33 cleavage, we used an *in silico* molecular weight prediction approach by comparing the sizes of Cp1-Cp3 estimated by Western blot to size predictions of amino acid sequences of various lengths from the C-terminal end. From this, we predicted approximate regions in the p33 sequence where potential cleavage may occur (Figure 1A; a schematic overview of the peptide products may also be found in Figure 8B). The LC–MS analyses did not show high enough resolution to identify proteolytic cleavage sites. Thus, the exact cleavage sites and sizes of the peptides could not be determined.

### 3.6 The hydrophobic domain is crucial for correct processing into peptides and directing peptides to a high membrane concentration

**Figure 9:**
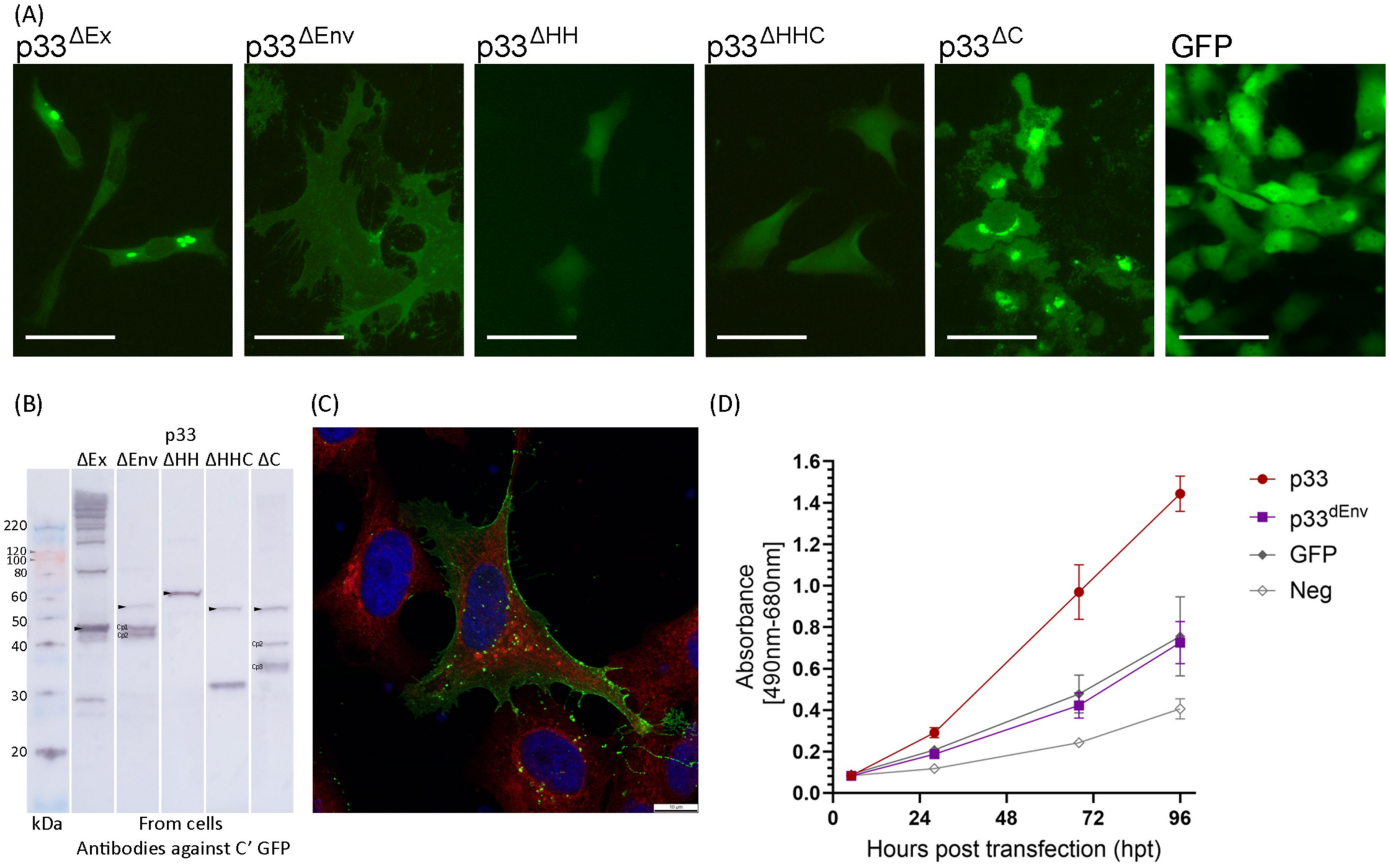
*In vitro* expression of p33 deletion variants in EPC cells, to characterize C-terminal end including hydrophobic domain. **(A)** Example of the characteristic appearance of p33 variants expressed with deletions related to studies of the C-terminal end/domain of high hydrophobicity (see text, Figure 1 for details), all with fluorescence from the C-terminal GFP tag. Cells expressing GFP only are included for comparative purposes. All imaging is performed at 2–3 dpt, excluding p33^ΔHH^, which was imaged at 5 dpt. Scale bars equal 50 μm. **(B)** Western blot studies of protein harvested from cells expressing p33 deletion mutants, with detection using antibodies against the GFP tag. The size of the full-size protein product is indicated by an arrowhead for full-length p33 mutants (corrected for deletion in the mutant) and main peptide products (Cp1-3) as discovered from the p33 reference are indicated (corrected for deletion as applicable) when present. Please refer to Figure 6 for comparative purposes. A mix of SeeBlue™ Plus2 Pre-stained Protein Standard (blue/orange bands) and MagicMark™ XP Western Protein Standard (gray bands) is used for size comparison in kilodaltons (kDa). Band size is given for MagicMark™, and gray arrowheads are added to clarify the visibility of weak 100 and 120 kDa bands. **(C)** High-resolution confocal image of a cell expressing p33^ΔEnv^ showing a high concentration of the expressed mutant and/or peptides with membrane localization from the C-terminal GFP tag. Red fluorescence indicates WGA-stained cell membranes, including high specificity for membranes of the Golgi, with higher intensity close to the nucleus. Blue staining indicates Hoechst-stained nuclei. Imaging is performed at 3 dpt. Scale bars equal 10 μm. **(D)** Lactate dehydrogenase (LDH) assay measuring the release of the cytosolic enzyme LDH into the surrounding cell culture media as an indicator of plasma membrane damage for p33^ΔEnv^ compared to p33 (both with GFP tags as described in the text) and also to cells expressing GFP only or a negative cell transfection control (all as described previously in Figure 2C). The absorbance from p33^ΔEnv^-expressing cells increases significantly over time (*p* < 0.001) but remains at indiscernible levels compared to GFP control cells. Representative images from microscopy of the cell cultures with phase contrast indicating the degree of CPE and parallel fluorescence microscopy showing protein expression from the GFP tag may be found in Supplementary Figure S2C.

We next focused on the C-terminal part of p33, which includes a domain with high hydrophobicity predicted to be transmembrane (Figure 1A). The approximate regions for proteolytic cleavage sites suggest that all three C-terminal peptide variants, Cp1-3, will include the hydrophobic domain (Figure 1A). As precise cleavage sites have not been defined in this work, we chose to study the importance of the C-terminal regions by constructing deletion variants based on the known amino acid sequence and domain characteristics (Figures 1A,D). In p33^ΔEx^, residues 60–204 were deleted (Δ60-204), representing the majority of the stretch from the signal sequence to the putative transmembrane domain, i.e., the part predicted to be extracellular (Figure 1D). Thus, only the signal sequence and a minor portion of the N-terminal end of the chemokine-like domain, along with the hydrophobic domain at the C-terminal end of p33, were expressed. All three cleavage sites are likely within the deleted region (Figures 1A,D). Transfected cells showed fluorescence from the expressed protein in the cytoplasm, including perinuclear and Golgi locations, and no phenotypic changes were observed (Figure 9A). As expected, no processed peptide products were seen by Western blot (Figure 9B). However, additional bands were observed, corresponding in size to multimers of two, three, and four copies, as well as several product bands above 220 kDa (Figure 9B), in addition to a band that may represent the GFP tag alone, as seen for other variants.

**Figure 10:**
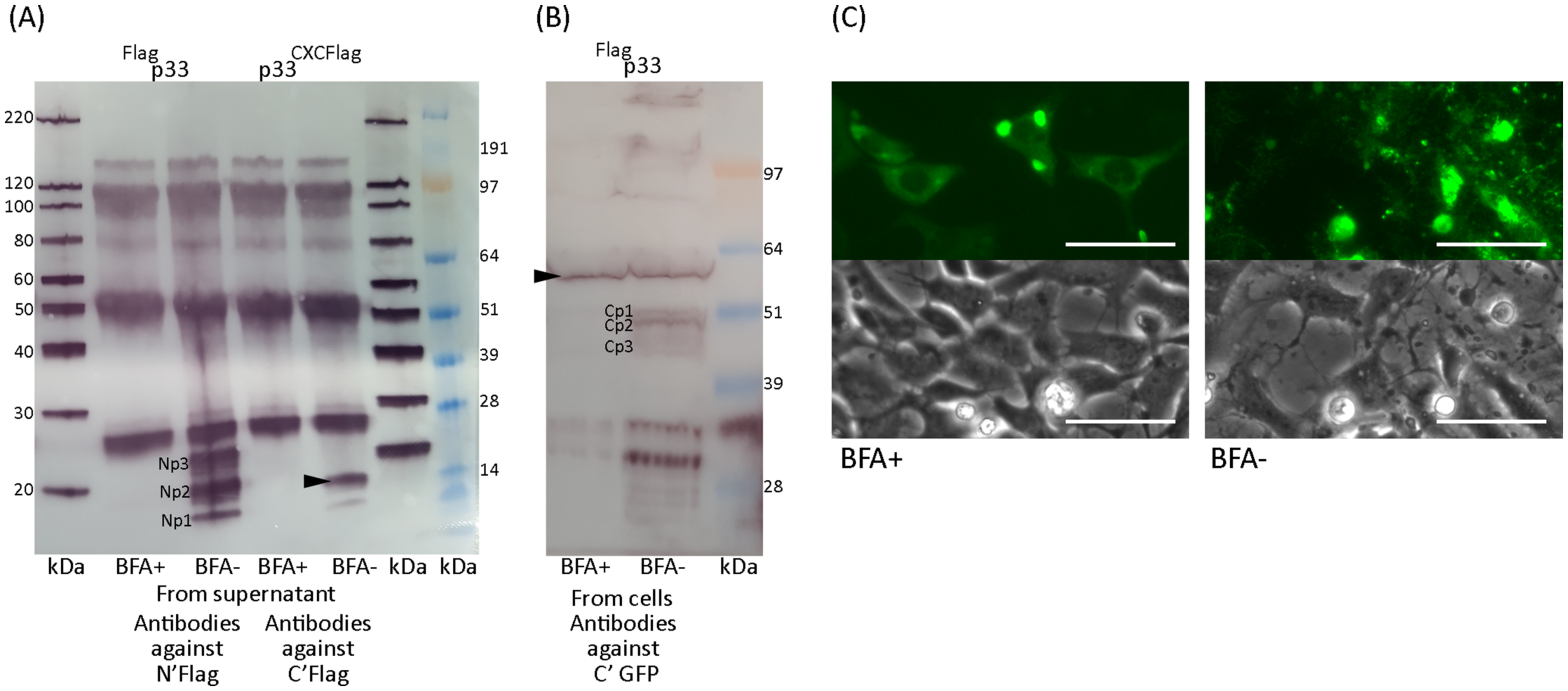
Effect of processing and secretion using Brefeldin A. **(A)** Western blot of peptides detected in the supernatant after full-length ^Flag^p33 or chemokine-like peptide p33_CXCFlag_ expressed from EPC cells in the presence of Brefeldin A (BFA+) or under normal conditions (BFA–). Chemokine-like peptides consistent with Np1-3 and p33^CXCFlag^ (arrowhead) are detected using anti-Flag antibodies, only under normal conditions. **(B)** Western blot of full-length protein and peptides detected in cells after full-length ^Flag^p33 expressed in EPC cells in the presence of Brefeldin A or under normal conditions. Full-length p33 (arrowhead) is detected under both conditions, while C-terminal peptides are not detected in the presence of Brefeldin A. Full-length p33 and C-terminal peptides are both detected using antibodies specific to the C-terminal GFP tag. **(C)** Microscopy studies of EPC cells transfected to express p33 in the presence of Brefeldin A or under normal conditions. Images from fluorescent microscopy are shown alongside a parallel image using phase contrast of the cell monolayer (3 dpt), showing that the cytotoxic phenotype is inhibited in the presence of Brefeldin A. Scale bars equal 50 μm.

A second deletion variant, p33^ΔEnv^ (Δ147–197), included the N-terminal half with a chemokine-like domain, an intact high hydrophobicity region, and a C-terminal end. This variant was originally constructed to test the effect of deleting the portion with weak homology to the HIV Env protein. After the proteolytic cleavage into sub-peptides was revealed, predictions indicated that the first and possibly second cleavage sites would still be present in p33^ΔEnv^, while the third was deleted (Figure 1D). Transfected cells exhibited a phenotype distinct from both normal cells and cells expressing control proteins, as well as from the cytotoxic phenotype. The cells were enlarged and displayed some cellular protrusions. Measurements of the area of EPC cells expressing p33^ΔEnv^ showed a mean cell surface area 2.5 times larger than that of cells expressing other proteins. Additionally, p33^ΔEnv^-expressing cells exhibited greater variation in size (measurement details and example images may be found in Supplementary Data Sheet S1). Fluorescence from the GFP tag was evenly distributed over the entire cell body, with clearly defined cell boundaries and indiscernible nuclei (Figure 9A, p33^ΔEnv^). This pattern contrasts with the typical distribution of cytoplasmic and nuclear proteins, which generally exhibit higher fluorescence intensity at the cell center that gradually diffuses toward the periphery (Figure 9A, GFP). The observed uniform distribution suggests a localization of GFP-tagged p33^ΔEnv^ components in relation to the plasma membrane. This was confirmed by confocal imaging, which showed a high concentration of fluorescent protein outlining the perimeter of the cell (Figure 9C). Western blot analysis revealed a low concentration of full-length mutant and a high concentration of two shorter products with sizes corresponding to Cp1 and Cp2 (Figure 9B). This is consistent with the presence of the first and second proteolytic sites and the deletion of the third. As this variant showed a phenotype different from the cytotoxic type but also different from normal cells, it was included in the LDH assays, which showed that LDH release levels were comparable to those found for general recombinant protein expression control (i.e., GFP-expressing cells) (Figure 9D), indicating no cytotoxicity specific to the expression of p33^ΔEnv^.

p33^ΔHH^ and p33^ΔHHC^ (Figure 1D) had the hydrophobic domain deleted (specifically residues 205–227 or, along with the remaining C-terminal end, residues 205–302) and did not exhibit any phenotypic changes compared to the GFP-only control. Only a few cells showed fluorescence from the expressed protein (Figure 9A). Both variants produced only full-length products, indicating no further processing, except for a possible cleavage of the GFP tag at the C-terminal end, as a product of approximately 30 kDa was observed (Figure 9B). This indicates that the two variants with deletions of the hydrophobic domain lost the transmembrane anchor, which delayed or disrupted correct expression and/or localization, resulting in low expression efficiency and/or degradation due to misfolding.

However, p33^ΔC^, which retained the hydrophobic domain while deleting the following residues up to the C-terminal end (Δ238-302) (Figure 1D), exhibited a phenotype similar to that of full-length p33 (Figure 9A, p33^ΔC^). By Western blot, p33^ΔC^ showed processed peptide products (Figure 9B). These findings indicate that the hydrophobic domain may be essential, either directly or indirectly, for correct processing into peptides and the observed characteristics, and it is presumably a transmembrane domain. The C-terminal portion following the putative transmembrane end is of lesser importance for function.

### 3.7 Brefeldin A inhibits p33 processing and secretion

Results thus far indicate that processing of the p33 protein into smaller peptides is required for the cytotoxic phenotype. Additionally, the p33^CXCFlag^ chemokine-like peptide induces a cytotoxic phenotype and is secreted from the cells. At what stage the processing of p33 occurs following translation remains unclear. To study this, we tested the effect of Brefeldin A (BFA), a known inhibitor of ER to Golgi transport. We used Western blots to characterize the protein fraction harvested from cell monolayers and their medium supernatants of treated (BFA+) or non-treated cells (BFA-). In the presence of BFA, the smaller peptides were not observed in cells and were not secreted into the supernatant (Figures 10A,B). Furthermore, Western blot analysis showed that BFA omitted detection of the p33^CXCFlag^ variant in the supernatant (Figure 10A). Additionally, p33 or the p33^CXCFlag^ did not induce the cytotoxic phenotype in BFA+ cells, as shown by phase contrast and fluorescence imaging (Figure 10C). The cytoplasm exhibited diffuse fluorescence and dense perinuclear granules, and the perimeter of the cells was clearly outlined. This indicates that processing of p33 is required for cytotoxicity, and inhibition of p33^CXCFlag^ transport through the Golgi and secretion has an inhibitory effect on cytotoxicity.

### 3.8 Single amino acid differences in wild-type p33 variants affect functional characteristics

**Figure 11:**
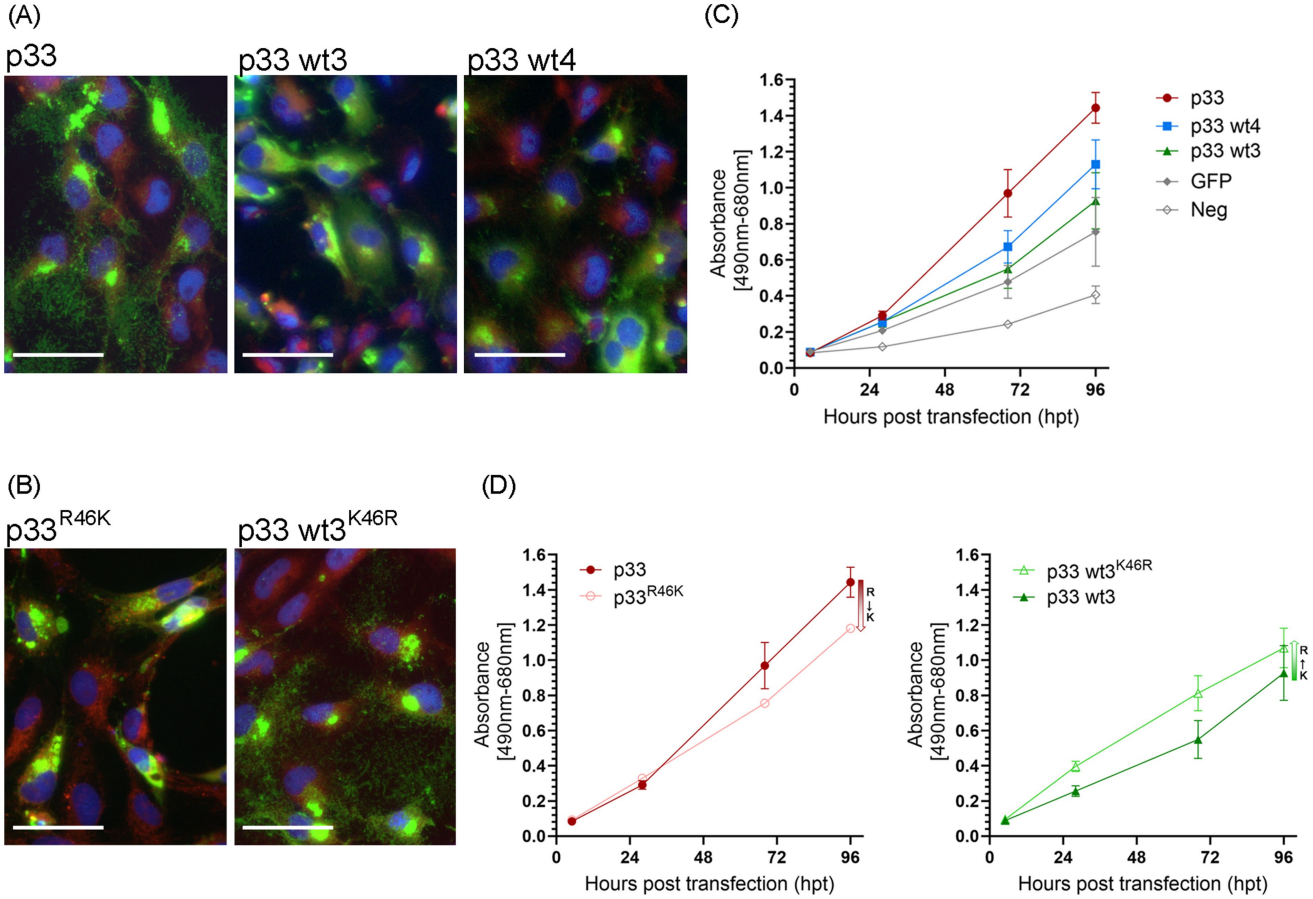
Single amino acid residues of importance in the p33 cytotoxic phenotype. **(A)** Example of the characteristic appearance of fluorescence from the expression of p33 wt3 and wt4 in EPC cells, resulting in low toxicity, with discernible cell cytoplasm and nuclei, and protein expression seen within the boundaries of the cell body. Images were captured at 3 dpt but reflect characteristic examples at any time point. Scale bar = 50 μm. **(B)** p33^R46K^ variant results in loss of function, i.e., an apparent silencing of the cytotoxic phenotype of the reference p33 protein. Inversely, the wt3^K46R^ variant gains function as the expressed protein shows a more cytotoxic phenotype compared to wt3. Cell membranes are stained red using CellMask™ Orange plasma membrane stain, and nuclei are stained blue using Hoechst. Scale bar = 50 μm. **(C,D)** Lactate dehydrogenase (LDH) assay measuring the release of the cytosolic enzyme LDH into the surrounding cell culture media as an indicator of plasma membrane damage. Absorbance was used to quantify LDH release over time. **(C)** Comparison of p33 wt3 and wt4. Absorbance from cells expressing both wt3 and wt4 increases significantly over time but at a slower rate than observed in p33-expressing cells (*p* < 0.007). For wt4, the difference compared to GFP control cells is statistically significant (*p* = 0.014), while there is no statistical difference between p33 wt3 and wt4 (*p* = 0.185). **(D)** Effect of residue 46 substitutions. Absorbance of cells expressing reference p33 compared to its p33^R46K^ variant (left) and p33 wt3 compared to its p33 wt3^K46R^ variant (right). LDH release increased significantly over time in all variants. However, substituting R with K at residue 46 reduced the rate of LDH release (*p* = 0.001), whereas substituting K with R increased the rate (*p* = 0.031). All p33 variants are with GFP tags as described in the text. Cells expressing GFP only or negative cell transfection were used as controls (all as described previously in Figure 2C). Representative images from microscopy of the cell cultures used in LDH analyses in **(C,D)**, with phase contrast indicating the degree of CPE and parallel fluorescence microscopy showing protein expression from the GFP tag, may be found in Supplementary Figures S2D,E, respectively.

Variants of p33 have previously been identified by sequencing field strains of PMCV from clinical disease outbreaks (Amono et al., 2024). This information was used to select 11 variants of p33, for which ORF3 was cloned and the p33 variant expressed (wt1-11, Table 1A) in cell cultures. The cellular phenotypes post transfection were compared to the p33 protein described above as a reference (p33). Three variants (wt3, 4, and 10) resulted in a less cytotoxic phenotype (shown for wt 3 and 4 in Figure 11A). LDH release was measured in parallel for wt3 and wt4 (Figure 11C) and was significantly reduced for both. Expression of wt3 indicated the least phenotypic changes compared to normal cells, and LDH levels released were not significantly distinguishable from GFP-expressing cells (Figure 11C). No shared amino acid substitutions were found among these three less cytotoxic variants (Table 1A). Wt4 has several radical substitutions in or in close vicinity to the C-terminal *α*-helix of the chemokine-like domain, in addition to one conservative substitution in the hydrophobic domain and one conservative substitution toward the C-terminal end (Table 1A). Wt10 has two radical substitutions: one (E_48_G) preceding the *β*1-strand of the chemokine-like domain and one in the hydrophobic domain (I_227_T, Table 1A). Wt3 has a conservative substitution, i.e., a change in the N-terminal amino acid in the chemokine-like domain (V_22_I) immediately following the signal sequence, a conservative substitution in the predicted small helix preceding the β-strands in the chemokine-like domain (R_46_K), and a radical change toward the C-terminal end (E_243_Q, Table 1A). The V_22_I and E_243_Q substitutions are the only substitutions in wt8, a variant giving a phenotype comparable to p33 reference variant. Based on this, we infer that the R_46_K substitution in the small helix of the chemokine-like domain is particularly important for the change in functional characteristics. Notably, the substitution found in the chemokine-like domain of wt10 (E_48_G) is close to this position and may hold similar importance. To evaluate the importance of the R_46_K substitution, we conducted a gain/loss of function study. A variant based on the reference p33 was generated, including the R_46_K substitution. p33 variants with R_46_G and R_46_E were also included, as Sanger sequencing of wt3 indicated a mix of variants (Table 1). In parallel, we prepared variants for a possible gain of function: wt3 K_46_R, K_46_G, and K_46_E. Microscopy and LDH assays showed that the R_46_K substitution in reference p33 reduced cytotoxicity. In contrast, the K_46_R substitution in p33 wt3 resulted in increased cytotoxicity (Figures 11B,D; Table 1B). These findings highlight the critical role of residue 46 in mediating cytotoxicity, with R being more functionally significant for cytotoxicity than K, G, or E. Wt1-6 were also analyzed for changes in proteolysis by Western blot, all resulting in the smaller peptide products as described for reference p33 (Supplementary Figure S5).

**Table 1:** Overview of p33 single substitution mutants found in the field (wt1-11) **(A)** and recombinant mutants to study gain and loss of cytotoxic expression characteristics in reference p33 and wt3 **(B)**.

	Chemokine-like											Hydrophobic						Residue
	22	24	**46**	**48**	**60**	65	78	84	87	97	114	**205**	**222**	227	242	243	259	Reference
	V	M	R	E	G	I	E	I	K	R	F	M	V	I	A	E	R
**(A) Wild type variants**																	
Wt1*	.	.	.	.	.	.	.	.	.	.	.	.	.	.	.	.	.
Wt2	.	.	.	.	.	.	.	.	.	.	.	.	.	.	.	Q	.
Wt3**	I	.	K	.	.	.	.	.	.	.	.	.	.	.	.	Q	.
Wt4	.	.	.	.	R	.	G	V	Q	Q	.	.	I	.	V	.	.
Wt5	.	.	.	.	.	.	.	.	.	.	L	.	.	.	.	.	.
Wt6	.	I	.	.	.	.	.	.	.	.	.	T	.	.	.	.	.
Wt7	.	I	.	.	.	.	.	.	.	.	.	T	.	.	.	.	H
Wt8	I	.	.	.	.	.	.	.	.	.	.	.	.	.	.	Q	.
Wt9	I	.	.	.	.	V	.	.	.	.	.	.	.	.	.	.	.
Wt10	.	.	.	G	.	.	.	.	.	.	.	.	.	T	.	.	.
Wt11*	.	.	.	.	.	.	.	.	.	.	.	.	.	.	.	.	.

(B) Recombinant mutants**																	
Ref	.	.	.	.	.	.	.	.	.	.	.	.	.	.	.	.	.
Ref R_46_K	.	.	K	.	.	.	.	.	.	.	.	.	.	.	.	.	.
Ref R_46_G	.	.	G	.	.	.	.	.	.	.	.	.	.	.	.	.	.
Ref R_46_E	.	.	E	.	.	.	.	.	.	.	.	.	.	.	.	.	.
Wt3	I	.	K	.	.	.	.	.	.	.	.	.	.	.	.	Q	.
Wt3 K_46_R	I	.	.	.	.	.	.	.	.	.	.	.	.	.	.	Q	.
Wt3 K_46_G	I	.	G	.	.	.	.	.	.	.	.	.	.	.	.	Q	.
Wt3 K_46_E	I	.	E	.	.	.	.	.	.	.	.	.	.	.	.	Q	.

The naming is given with color coding, where red indicates a resulting cytotoxic phenotype and green indicates low or no resulting cytotoxicity (see text for details). *Wt1 and Wt11 are equal to reference p33 but are included as controls originating from different sources. Wt11 has two silent nucleotide mutations compared with reference. **In Sanger sequencing chromatograms, Wt 3 was found with a double peak for one nucleotide in the codon representing amino acid 22, resulting in either V or I, and for two nucleotides in the codon for amino acid 46, representing R (as in ref), K (as in expressed protein variant), but also G or E. The resulting cloned variant is as indicated, but recombinantly produced variants, including G or E in residue 46, were included in **(B)**.

## 4 Discussion

Here, we have shown that *in vitro* recombinant expression of PMCV p33 resulted in processing into smaller peptides, which induced a cytotoxic phenotype in transfected cells. Furthermore, N-terminal chemokine-like peptides were secreted into the extracellular environment through the ER and Golgi complex, and the expression of these peptides separately resulted in cytotoxicity. The C-terminal counterpart peptides included a hydrophobic region, which may dock the full-length protein or peptides into membranes and be directly or indirectly important for correct peptide processing. N-terminal peptides of three different sizes are secreted, while three differently sized C-terminal putative counterparts remain intracellular. The specific cleavage sites have not been identified; thus, the exact size of the peptides and the reason for the observation of three different sizes of each peptide are not known.

Viral chemokines, also known as “virokines,” have predominantly been identified in large double-stranded DNA viruses, including baculoviruses, poxviruses, ascoviruses, and herpesviruses (reviewed in Murphy, 2001). These virokines are believed to originate from host chemokine genes that were acquired and subsequently adapted during viral evolution. In addition to authentic chemokines encoded by viruses, certain viral proteins or peptides with structural features distinct from classical chemokines, such as HIV-1 Tat, have been shown to interact with chemokine receptors and modulate their function. Virokines and viral chemokine mimicry can be classified based on their functional roles, which include acting as anti-chemokines, facilitating viral entry into host cells, promoting cell growth, inducing angiogenesis, and serving as leukocyte chemoattractants. By interfering with host chemokine signaling, these viral factors play a crucial role in immune evasion, facilitating viral persistence and pathogenesis by subverting host defense mechanisms and disrupting cellular communication (Murphy, 2001). Our results demonstrate that the chemokine-like domain is secreted into the extracellular compartment, and the result is exerts cytotoxic effects on cells. While several virokines induce cytotoxicity by activating host immune cells, we found no comparable examples of virokines causing direct cytotoxicity in an *in vitro* system lacking immune cell components. Secreted viral cytotoxic proteins have been previously described, including rotavirus non-structural protein 4 (NSP4) enterotoxin and HIV-1 Tat. Both are expressed in infected cells, secreted into the extracellular environment, and impact both infected and uninfected cells (Zhang et al., 2000; Jagannath et al., 2006; Seo et al., 2008; Ajasin and Eugenin, 2020). NSP4 engages integrins as cell-specific receptors (Dong et al., 1997; Seo et al., 2008). Tat, in contrast, interacts with a broad range of receptors, facilitating uptake by immune, nervous, and cardiovascular cells. Notably, Tat exhibits chemokine mimicry, dysregulating host immune responses and contributing to chronic inflammation associated with HIV comorbidities (Albini et al., 1998; Mishra et al., 2008; Ajasin and Eugenin, 2020). However, if similar receptor usage may relate to the functional effect of the PMCV chemokine-like molecule remains to be elucidated.

Blocking the ER–Golgi transport of p33 inhibits processing into peptides and prevents the associated cytotoxic phenotype in transfected cells, supporting the notion that processing is needed for cytotoxicity. The chemokine-like peptide (p33^CXCFlag^) exhibits cytotoxic activity when expressed on its own, although at a reduced level compared to full-length p33. Furthermore, deletion of a major portion of the chemokine-like region (p33^ΔCXC^) abolishes cytotoxicity. These findings suggest that the N-terminal chemokine-like peptides may exert cytotoxic effects independently. However, the underlying mechanisms remain unclear and warrant further investigation, specifically whether the cytotoxicity arises during the secretion process or is mediated by the secreted product, and whether this effect is general or restricted to cells expressing the peptide. Additionally, potential cooperative or modulatory interactions involving one or more variants of the C-terminal peptide, particularly its hydrophobic region, should also be explored.

PMCV infection causes initial inflammation in the atrium and spongy part of the ventricle of Atlantic salmon (Fritsvold et al., 2009; Haugland et al., 2011), resulting in severe myocardial necrosis in both heart compartments and leaving few intact cardiomyocytes, ultimately described as necrotizing myocarditis (Fritsvold et al., 2009). The viral genome has been detected *in situ* in degenerate and necrotic myocytes (Haugland et al., 2011; Fritsvold et al., 2022) and by antibodies against p33 (Fritsvold et al., 2021). *In vitro*, the cytotoxicity is indicated to be linked to both apoptotic and necrotic processes, possibly in sequence. Based on the cytotoxic properties of p33 *in vitro* and the degeneration and necrosis of cardiomyocytes *in vivo*, it is likely that p33 contributes to the pathogenesis of myocardial necrosis. The extent to which p33 and/or peptides and their cytotoxic phenotype play a role in the spread of the virus from infected cells and constitute an essential mechanism for the release of viral progeny remains to be proven.

The functional characteristics of the C-terminal peptides remain unclear. We have not yet been able to define precise cleavage sites experimentally or through *in silico* predictions, and consequently, functional expression assays with correctly sized peptides have not been possible. The hydrophobic region appears to be direct or indirect importance for correct processing into peptides and directing peptides for membrane localization. Our present *in silico* analyses add predictions describing a N_out_/C_in_ type I topology in the membrane. Additional detailed protein sequence analyses show that the hydrophobic domain is immediately followed by several lysines and an arginine. These characteristics align with Class 1A viroporins, which are described as a diverse group of small, hydrophobic transmembrane proteins encoded by viruses, generally characterized by an amphipathic transmembrane domain followed by a cluster of basic residues, such as lysine or arginine (Xia et al., 2022). Viroporins oligomerize within host cell membranes to form hydrophilic pores, disrupting various physiological properties, and may play roles throughout the viral life cycle, including entry/penetration, genome replication, and progeny virus release (Xia et al., 2022). Fusion-associated small transmembrane (FAST) proteins constitute one specific type of Class IA viroporin in non-enveloped viruses, i.e., orthoreoviruses such as avian orthoreovirus (ARV), and several aquareoviruses, which all infect fish (Duncan, 2019). Interestingly, FAST-like proteins are also indicated to be expressed from the pistolvirus CLuTLV (Sandlund et al., 2021) and tentative pistolvirus SBTLV (Louboutin et al., 2023). FAST proteins are mainly characterized by their membrane fusogenic properties, which result in syncytium formation. However, they may also produce a cytolytic effect as the syncytia rupture when they reach a specific size, leading to bursts of viral progeny (Duncan, 2019).

The indications of a FAST-like protein encoded by other pistolviruses, together with the amino acid composition characteristics found in the C-terminal part of p33, support a hypothesis that PMCV p33 includes a FAST-like protein function. To further elucidate this, additional studies are needed to precisely define cleavage sites and peptide length, followed by experimental expression studies of individual peptides. These investigations will provide deeper insights into the functional role of p33.

The mechanisms by which wild-type p33 variants with a few amino acid substitutions influence the cytotoxic phenotype remain unclear. However, the loss or gain of function observed with single amino acid substitutions at residue 46 of the p33 reference and wt3 variants is intriguing. The substitution is considered conservative, and the residue is located in the predicted short helix preceding the three *β*-strands of the chemokine-like secreted peptide. Based on both amino acid sequence and predicted 3D structure (Figure 1A), this residue appears to be relatively distant from residues putatively involved in receptor-binding at the N-terminal end and N-loop/β3-strand, as indicated by homology to chemokines (Bhusal et al., 2020). Nonetheless, the results suggest that it does influence the functional properties of p33. While experimental proof of the receptor-binding residues within the p33 chemokine-like domain is still needed, indirect effects on receptor binding cannot be excluded. Additionally, amino acid substitutions in wt4 and wt10 were identified within the chemokine-like domain, along with further substitutions in the hydrophobic domain, which may or may not influence expression characteristics. Given that the cytotoxic effect is associated with membrane leakage, these p33 variants are likely to have a diverse impact on membrane integrity and may therefore define virus variants with different levels of virulence. Unfortunately, field data from CMS case populations or individual fish harboring PMCV variants with p33 wild-types that differ in functional properties, as described above, are insufficient to draw conclusions about corresponding effects on disease severity or viral loads in infected individuals.

Some classical totiviruses (family *Orthototiviridae*, infecting fungi) take advantage of proteins with cytotoxic characteristics (usually called killer toxins). These proteins are not encoded by the virus but by satellite dsRNA that use similar encapsidation to the virus to parasitize their replication processes (Schmitt and Breinig, 2006; Becker and Schmitt, 2017). These killer toxins are secreted and kill non-infected cells, while the secreting cells remain “immune.” They disrupt cell membranes, inhibit DNA synthesis, and are small. Proteolytic processing is required for their activity (Schmitt and Breinig, 2006; Becker and Schmitt, 2017). The small size and requirement for proteolytic activation for the cytotoxic effect we have shown for p33 could indicate that p33 results from the integration of a killer toxin gene from a parasitic satellite virus into the genome of an ancestral totivirus, which created the basis for horizontal transfer into more advanced hosts. However, the structural similarity to a secreted chemokine and a hydrophobic, putative transmembrane domain in the C-terminal peptide is inconsistent with the origin being a killer toxin. Additional studies would be required to elucidate the evolutionary origin of PMCV and its third ORF.

The reorganization of the order *Ghabrivirales* now incorporates new virus families, including *Artiviridae* with arthropod hosts and *Pistolviridae* with piscine hosts. Unlike the major virus families of the order, which primarily infect single-celled organisms, these newly classified families include viruses capable of infecting multicellular hosts. These viruses are characterized by the release of viral particles from infected cells and extracellular transmission (Current ICTV Taxonomy Release, 2023). The adaptation to multicellular hosts, which also exhibit more complex antiviral defense mechanisms, is reflected in additional viral coding sequences. These sequences likely confer the functional properties required to overcome advanced host defenses and enable efficient transmission (Poulos et al., 2006; Haugland et al., 2011; Dantas et al., 2016; Shao et al., 2021). For some of the artiviruses, it has been shown that the virus particle includes surface protrusions, which have not been observed in viruses infecting single-celled organisms within the virus order. For these viruses, additional coding sequences in the 5′-end of the genome encode the small protein fragments that constitute these protrusions (Tang et al., 2008; Shao et al., 2021). It has also been suggested that the chemokine-like part of PMCV p33 is a structural protein and may account for adhesion to a chemokine receptor, as seen in HIV (Haugland et al., 2011; Nibert and Takagi, 2013). Although it cannot be ruled out that p33 or peptide products may be a structural component of the virus, our findings suggest that it has a non-structural role during virus replication.

In summary, we provide novel insights into the multifunctional virus protein p33, which shows no overall homology to known viral proteins. However, specific peptide regions share structural or sequence similarities with chemokines (at the N-terminal end) or viroporins, such as FAST proteins (at the C-terminal end). The full-length protein p33 is likely to be anchored to the ER membrane, transported through the ER–Golgi pathway, and cleaved at multiple sites, possibly upon transfer from vesicles to plasma membranes. The resulting peptides are predicted to have distinct functional properties, including cytotoxic effects. Beyond enhancing our understanding of the genetic requirements for a dsRNA virus to transition from infecting single-celled organisms to more complex hosts, our findings on p33 may also inform the development of targeted strategies for combating CMS in farmed Atlantic salmon, a disease for which no vaccines are currently available.

## Data Availability

The datasets presented in this study can be found in online repositories. The names of the repository/repositories and accession number(s) can be found at: https://www.ncbi.nlm.nih.gov/genbank/, OQ615313–OQ615323.

## Supplementary material

Supplementary Figure S1 EPC cells expressing p33 with C-terminal GFP-tag at 1, 2, and 5 days post transfection (dpt) by fluorescence and phase contrast imaging. The images correspond to original images of Figure 2B, where it was cropped to show single cells expressing p33. Control cells expressing PMCV capsid with C-terminal GFP-tag and negative control cells (mock transfected with no plasmid addition) cells run in parallel are included for 5 dpt.

Supplementary Figure S2 EPC cells expressing the p33 variants from cell cultures analyzed in LDH-assay at first (28 hpt) and last (96 hpt) time points analyzed by parallel phase contrast and fluorescence imaging. **(A)** Images corresponding to in Figure 2C. **(B)** Images corresponding to assay in Figure 7C. **(C)** Images corresponding to assay in Figure 9D. **(D)** Images corresponding to assay in Figure 11C. **(E)** Images corresponding to assay in Figure 11D.

Supplementary Figure S3 CHH-1 cells expressing p33 with a C-terminal His-tag (p33His) visualized using IFAT with antiHis-antibodies as shown in Figure 3 and additionally Hoechst-stained nuclei of all cells indicating that the antiHis is specific to detecting p33^His^ and with no background staining of normal cells.

Supplementary Figure S4 CHH-1 cells expressing p33 at 1, 2, 3, and 6 dpt showing increasing condensed/fragmented nuclei correlating with increasing expression of p33, shown by parallel fluorescence imaging of green fluorescence from p33 C’ terminal GFP-tag, Hoechst stained nuclei in blue with increased number of condensed/fragmented nuclei (intense light blue) over time and also correlating with propidium iodide staining in red fluorescence.

Supplementary Figure S5 Western blot studies of protein harvested from EPC cells after recombinant expression of p33 wt1-6 with C-terminal GFP-tag. All p33 wt1-6 are processed into Cp1-3 and smaller peptide products similar to p33 reference (shown in Figure 6). Proteins are detected using antibodies against GFP-tag. SeeBlue^TM^ Plus2 and MagicMark™ XP Western Protein Standard is used for size comparison in kilodaltons (kDa).
