# Supplementary figures and images for: Defined domains and cleavage determine the diverse functions of piscine myocarditis virus p33 protein
Source: Front Microbiol. 2025 Sep 1;16:1633241. doi: 10.3389/fmicb.2025.1633241 (PMC12433951; doi:10.3389/fmicb.2025.1633241)

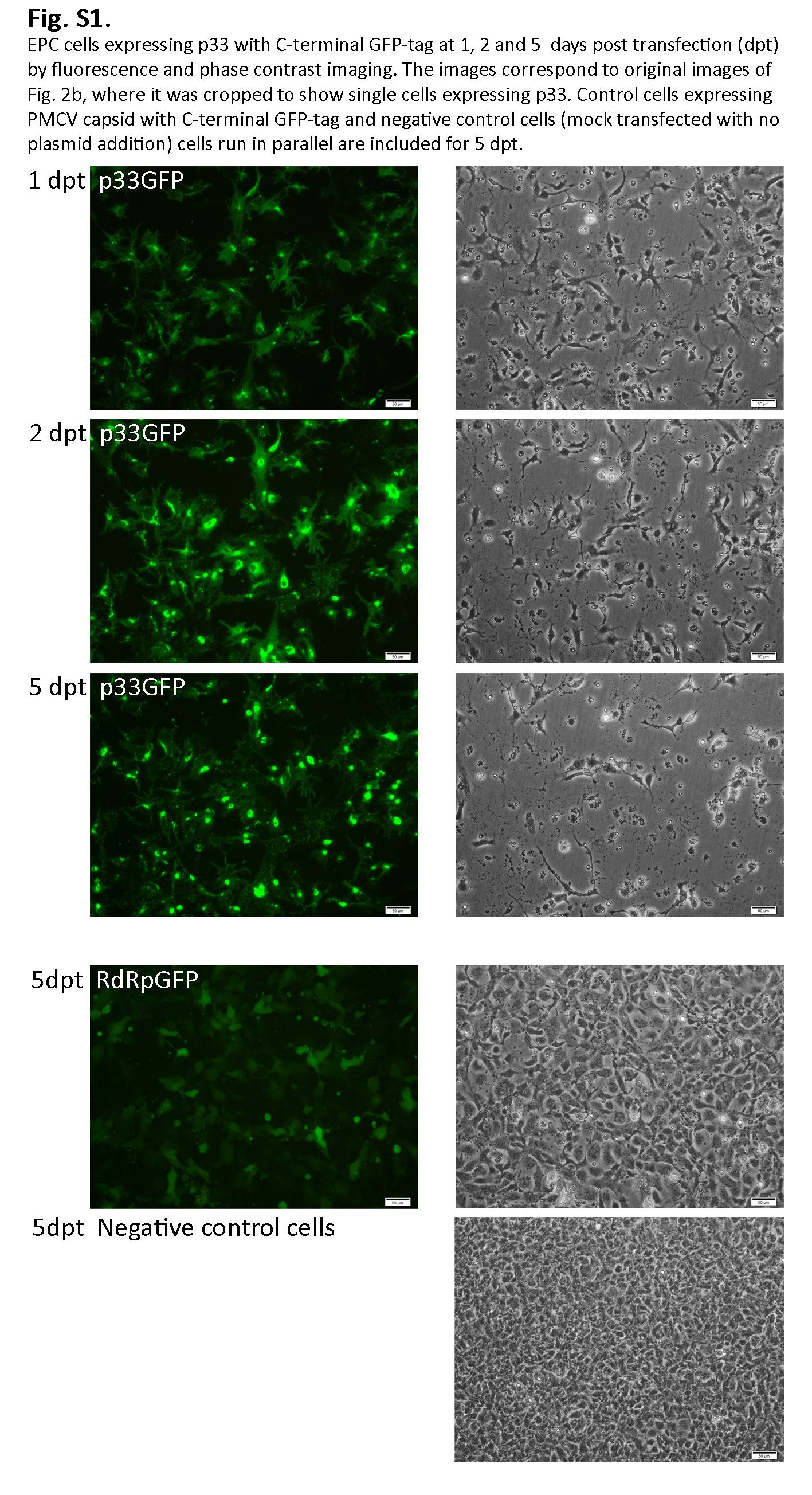

Supplement: Supplementary Figure S1 — EPC cells expressing p33 with C-terminal GFP-tag at 1, 2, and 5 days post transfection (dpt) by fluorescence and phase contrast imaging. The images correspond to original images of Figure 2B, where it was cropped to show single cells expressing p33. Control cells expressing PMCV capsid with C-terminal GFP-tag and negative control cells (mock transfected with no plasmid addition) cells run in parallel are included for 5 dpt. [file Image_1.TIF]

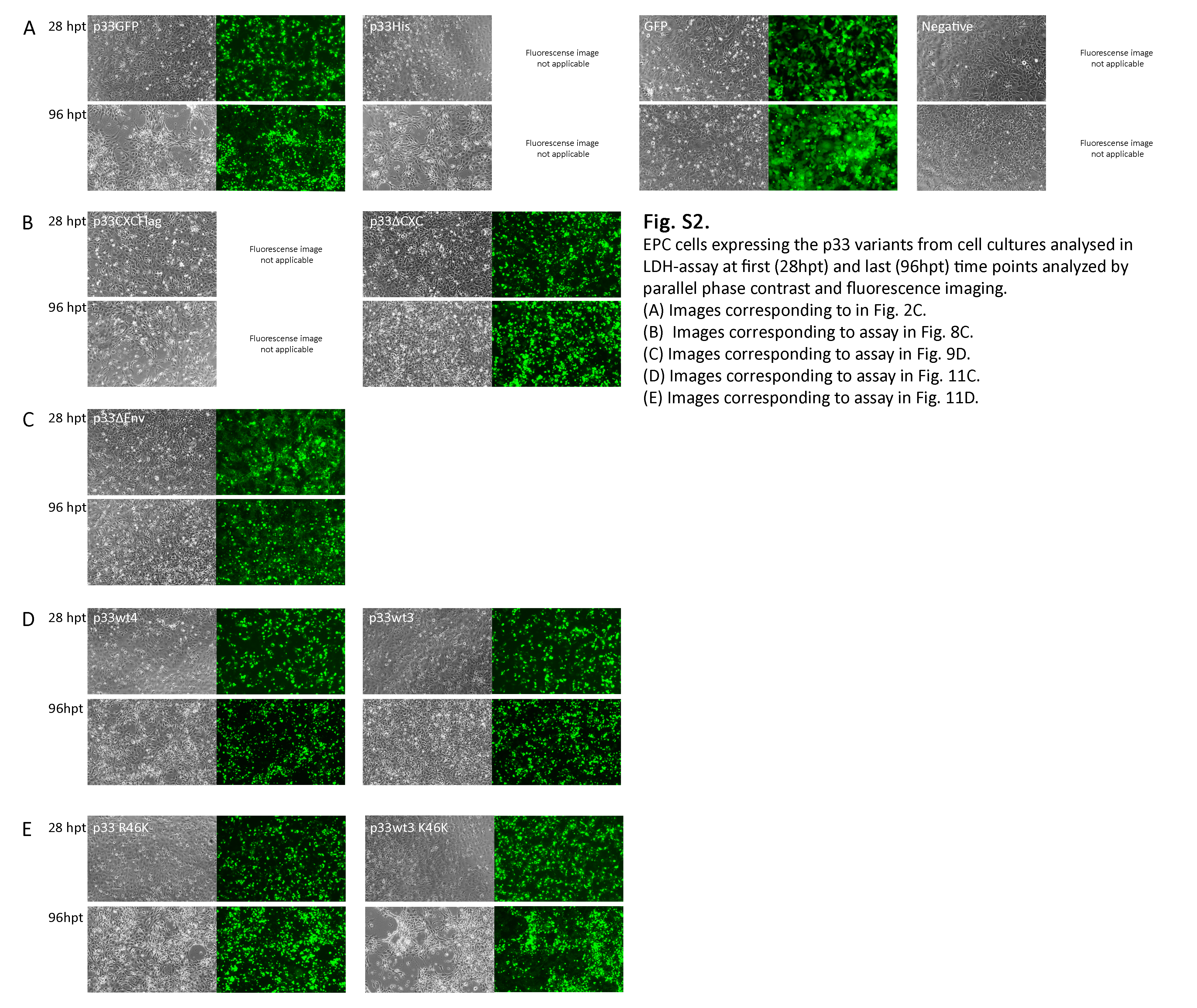

Supplement: Supplementary Figure S2 — EPC cells expressing the p33 variants from cell cultures analyzed in LDH-assay at first (28 hpt) and last (96 hpt) time points analyzed by parallel phase contrast and fluorescence imaging. (A) Images corresponding to in Figure 2C. (B) Images corresponding to assay in Figure 7C. (C) Images corresponding to assay in Figure 9D. (D) Images corresponding to assay in Figure 11C. (E) Images corresponding to assay in Figure 11D. [file Image_2.TIF]

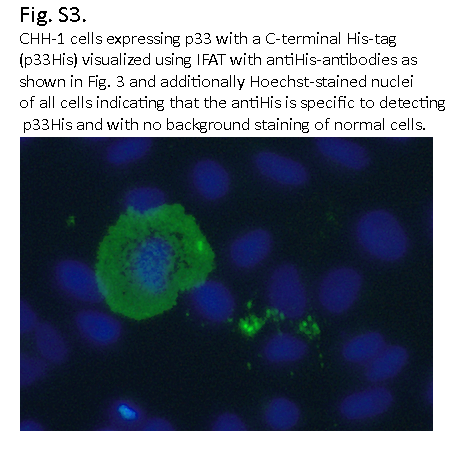

Supplement: Supplementary Figure S3 — CHH-1 cells expressing p33 with a C-terminal His-tag (p33His) visualized using IFAT with antiHis-antibodies as shown in Figure 3 and additionally Hoechst-stained nuclei of all cells indicating that the antiHis is specific to detecting p33His and with no background staining of normal cells. [file Image_3.TIF]

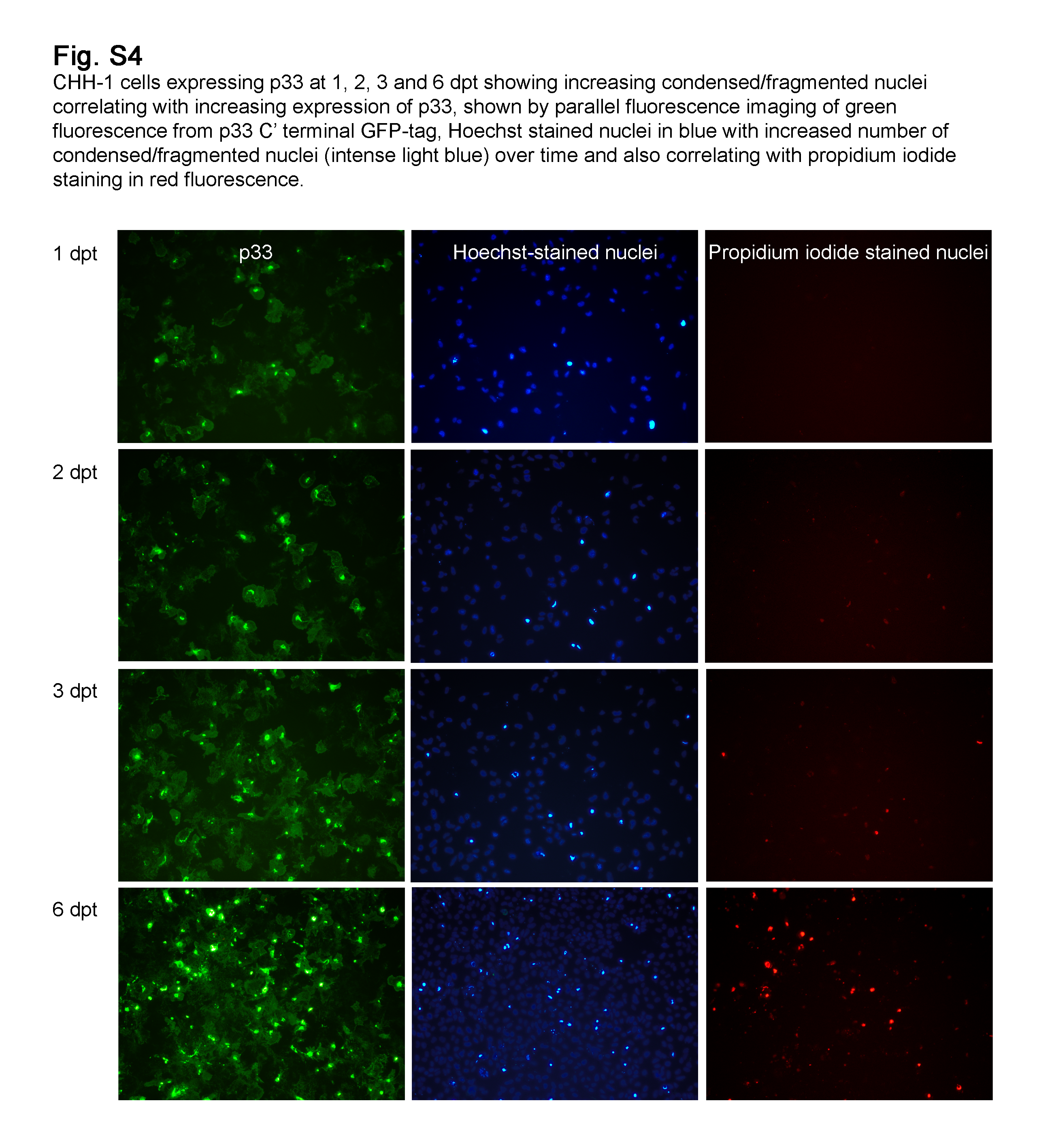

Supplement: Supplementary Figure S4 — CHH-1 cells expressing p33 at 1, 2, 3, and 6 dpt showing increasing condensed/fragmented nuclei correlating with increasing expression of p33, shown by parallel fluorescence imaging of green fluorescence from p33 C’ terminal GFP-tag, Hoechst stained nuclei in blue with increased number of condensed/fragmented nuclei (intense light blue) over time and also correlating with propidium iodide staining in red fluorescence. [file Image_4.TIF]

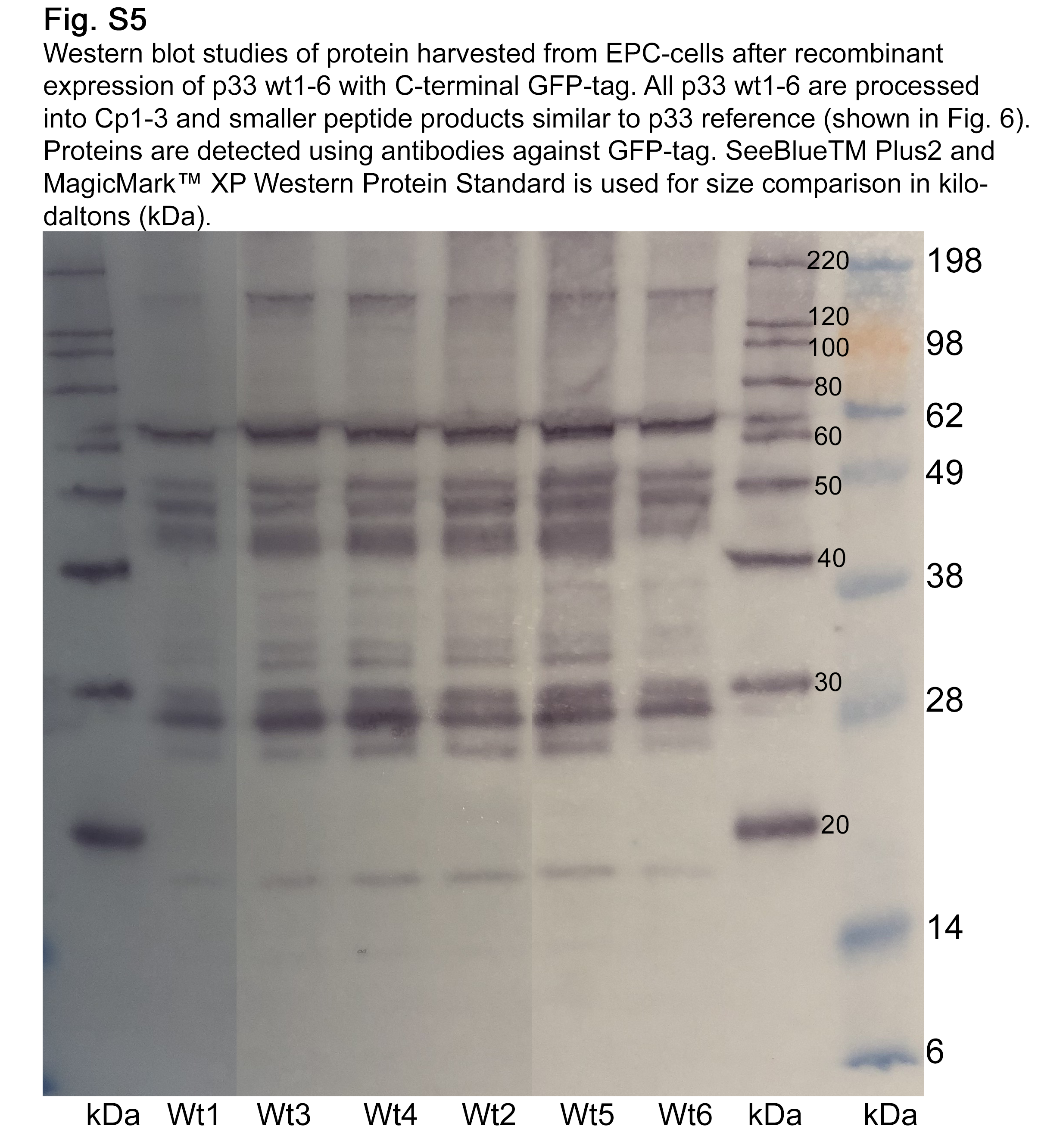

Supplement: Supplementary Figure S5 — Western blot studies of protein harvested from EPC cells after recombinant expression of p33 wt1-6 with C-terminal GFP-tag. All p33 wt1-6 are processed into Cp1-3 and smaller peptide products similar to p33 reference (shown in Figure 6). Proteins are detected using antibodies against GFP-tag. SeeBlueTM Plus2 and MagicMark™ XP Western Protein Standard is used for size comparison in kilodaltons (kDa). [file Image_5.TIF]
