# Supplementary material for: Defined domains and cleavage determine the diverse functions of piscine myocarditis virus p33 protein
Source: Front Microbiol. 2025 Sep 1;16:1633241. doi: 10.3389/fmicb.2025.1633241 (PMC12433951; doi:10.3389/fmicb.2025.1633241)

Supplementary Material S1

Cell and protein structure measurements using ImageJ

Method

Cell size or size of p33 protein structures measured as area of the cells or structures using digital images obtained through fluorescence or phase contrast imaging. Images of CHH-1 cells transfected to express PMCV capsid, RdRp or p33, all with C-terminal GFP tag, were analyzed using ImageJ.JS software (https://ij.imjoy.io/). All images originated from a single experimental transfection set up for expression of all three proteins and included one image from each of 2, 5 and 6 days post transfection (dpt). In addition, parallel phase contrast images of cells expressing capsid, RdRp or p33 at 2dpt were included to measure cells negative for protein expression (no visible fluorescence in parallel fluorescence image) were included. Cell size of cells expressing p33$^{\Delta Env}$ were similarly compared to PMCV capsid expressing cells using images of transfected EPC cells from a single experimental set up and included images from 1 and 2 dpt for p33$^{\Delta Env}$ and 7dpt for PMCV capsid.

From each image 8-11 cells or protein structures were measured. Multinucleated cells, partly detached cells or other cells deviating from the general characteristics of each cell culture, were not included. The sizes were measured using the ImageJ freehand selection tool and area of encircled object quantitatively measured as square pixels.

Results

p33 protein structures resulting from p33 expressing CHH-1 cells varies in size and may be larger than normal single cells

A quantitative measurement of the area for p33 protein structures compared to normal cells showed that the mean size of p33 protein structures was from 1.6-1.9 times larger than cells expressing other proteins or non-expressing cells (Fig. 1). p33 protein structures also had a higher variation in size compared to the normal cells (Fig. 1).

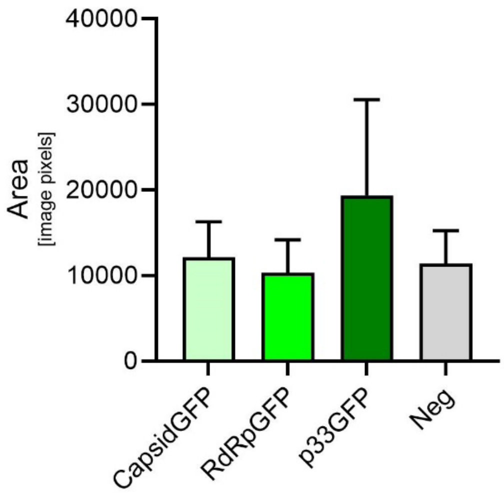

Fig. 1 - Comparison of p33 protein structure size to size of normal cells, i.e. cells expressing capsidGFP, RdRpGFP or cells with no apparent protein expression.

Example images of CHH-1 cells expressing p33 structures and parallel cell cultures expressing capsid protein is shown in Fig. 2.

A)

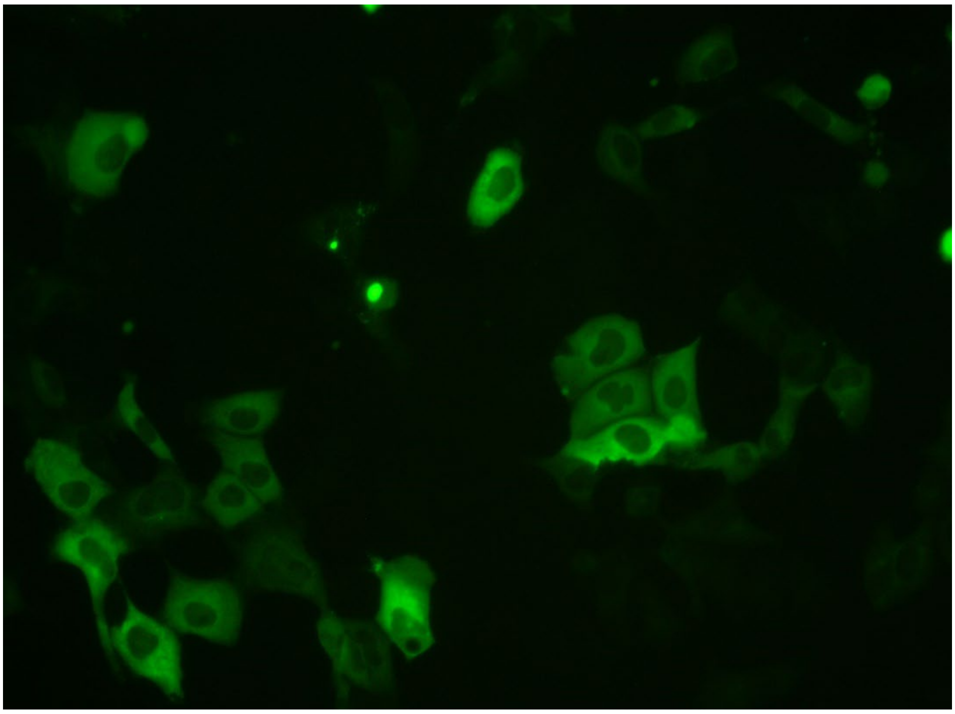

B)

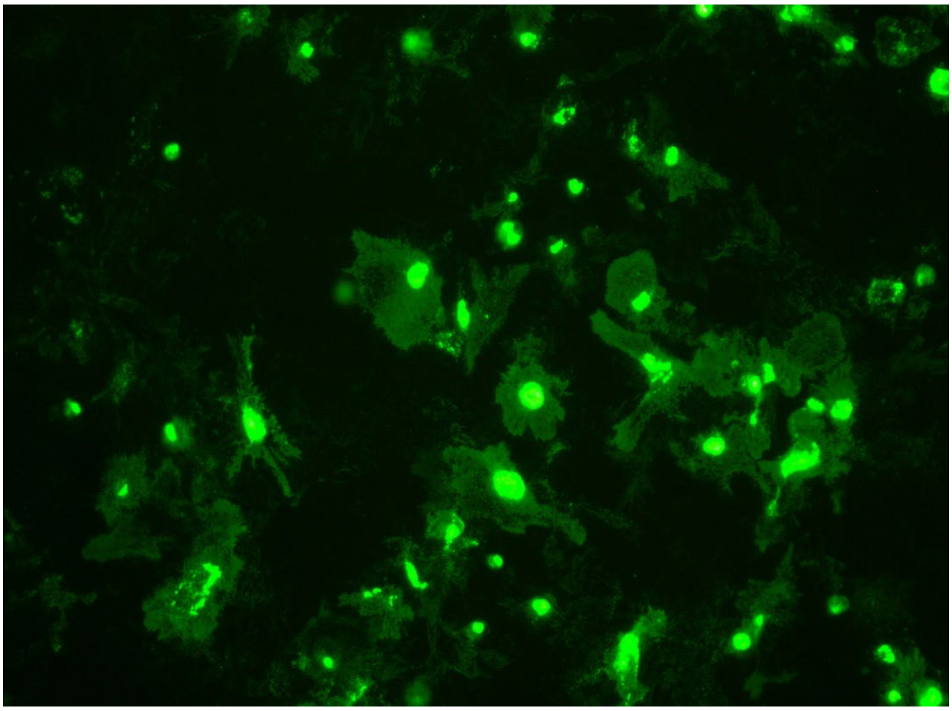

Fig. 2 – Fluorescent microscopy images of PMCV capsid (A) and p33 (B) expression in CHH-1 cells at 6 dpt. Expression of capsid is seen with cytoplasmic localization with and a nice outline of the perimeter of standard normal cells. Compared to capsid expressing cells in A) the p33 protein structures shown in B) deviates from normal cells both by morphology and variability in size.

p33$^{\Delta Env}$ expressing EPC cells varies in size and may be larger than normal single cells

A quantitative measurement of the area for p33$^{\Delta Env}$ expressing EPC cells compared to normal cells showed that the mean size of such cells was 2.5 times larger than cells expressing RdRp (Fig. 3). p33$^{\Delta Env}$ expressing EPC cells also had a higher variation in size compared to the normal cells (Fig. 3).

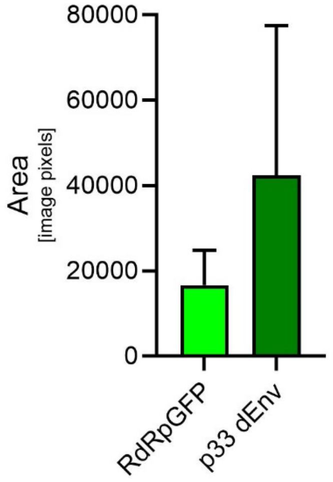

Fig. 3 - Comparison of size of p33$^{\Delta Env}$ expressing EPC cell to size of normal cells, i.e. cells expressing RdRpGFP.

Example images of EPC cells expressing p33$^{\Delta Env}$ and parallel cell cultures expressing RdRp protein is shown in Fig. 4.

Fig. 4 Fluorescent microscopy images of PMCV RdRp (A) and p33$^{\Delta Env}$ (B, C) expression in EPC cells at 7 dpt and 2dpt, respectively. Expression of RdRp is seen with cytoplasmic and nucleic localization and with a nice outline of the perimeter of standard normal cells. Compared to RdRp expressing cells in A) the p33$^{\Delta Env}$ expressing cells shown in B) and C) deviates from normal cells both by morphology and variability in size.

A)

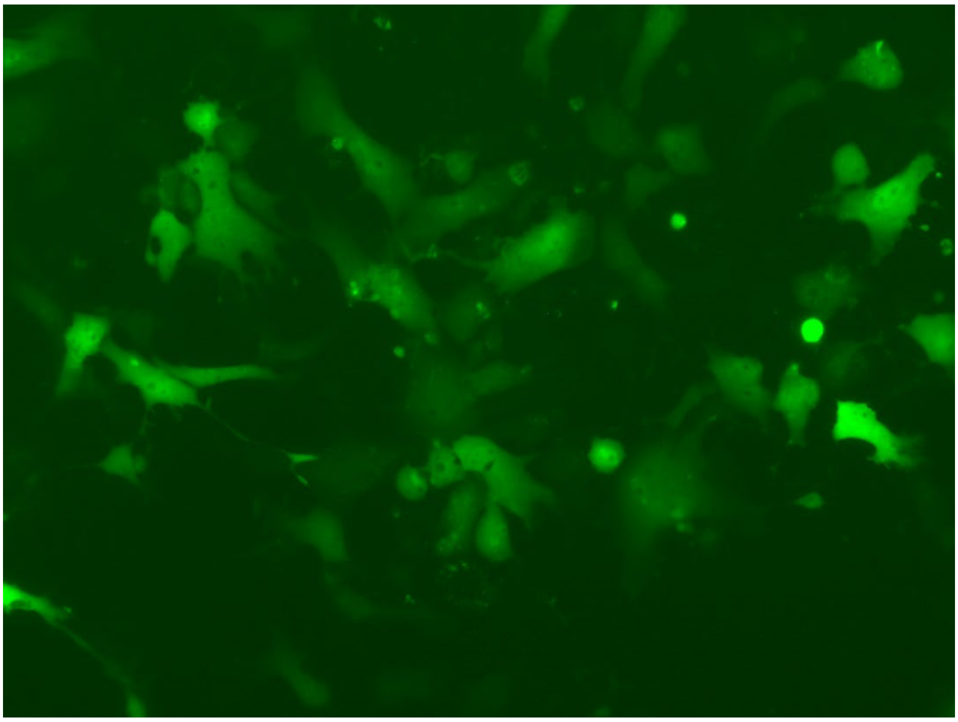

B)

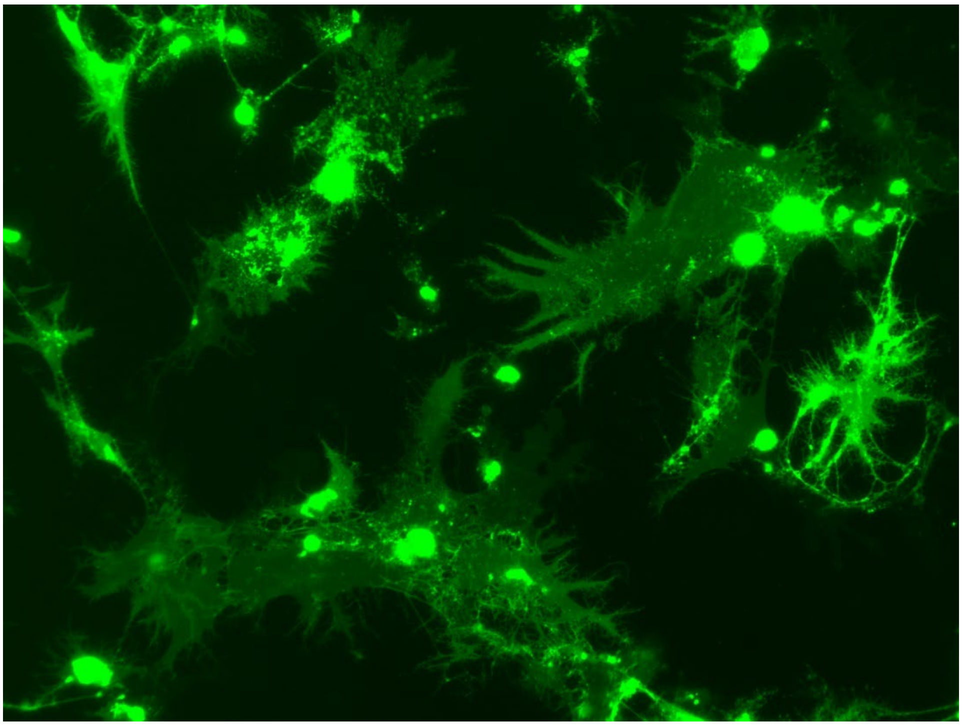

C)

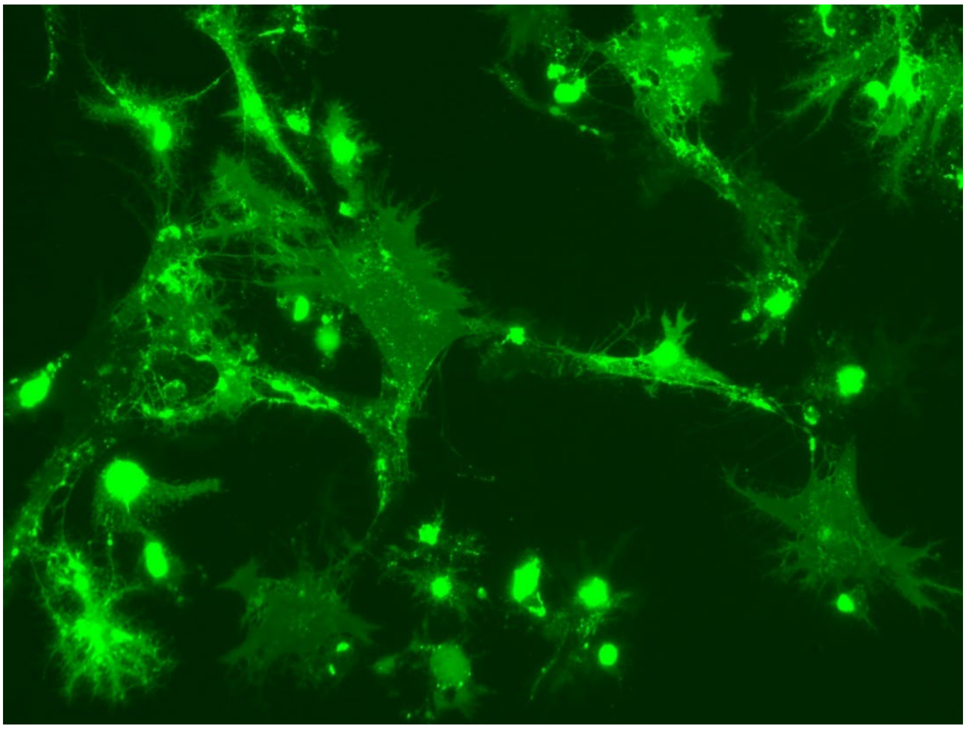

Supplement: Supplementary file 6 [file Data_Sheet_1.PDF]
