# Supplementary material for: Defined domains and cleavage determine the diverse functions of piscine myocarditis virus p33 protein
Source: Front Microbiol. 2025 Sep 1;16:1633241. doi: 10.3389/fmicb.2025.1633241 (PMC12433951; doi:10.3389/fmicb.2025.1633241)
Supplement: Supplementary file 7 [file Table_1.PDF]

Supplementary

Table S1

Primers used in construction of expression vector plasmids, sequencing and PCR. Restriction sites and nucleotides for introduction of His- or Flag-tag are color coded. Sequence non-specific to template due to 5’ overhang is underlined. Mutations to be introduced are shown in bold.

Cloning of PMCV ORFs into vector for expression with GFP-tag

Seq. origin	Amplicon	Forward-primer		Reverse-primer		Resulting product (protein variant)
**Cloning into pmaxFP-Green-N**						
AL V-708[a]	XhoI - ORF1 - HindIII	ORF1n-XhoI-F	**CTCGAG**CCAACTATGGAACCA	ORF1n-HindIII-R	**AAGCTT**TATATCTAAAAAATCTGCAGTTG	**pORF1green (Capsid)**
AL V-708[a]	XhoI - ORF2 - HindIII	ORF2-XhoI-F	GGCA**CTCGAG**GAATGATAACCAAAGCAT	ORF2n-HindIII-R	CCGG**AAGCTT**CTGTAATATCCTTTGCC	**pORF2green (RdRp)**
AL V-708[a]	XhoI - ORF3 - HindIII	ORF3-XhoI-F	GGCC**CTCGAG**GGATGTCAAACAAGATGAAGAGTTTT	ORF3n-HindIII-R	CCGG**AAGCTT**TAAGACTCCTACTCTGAA	**pORF3green (p33)**
Wt 1-11[b]	XhoI - ORF3^{wtX} - HindIII					**pORF3green^{wtX} (p33 wtX)**
**Cloning into pmaxFP-Green-C**						
AL V-708[c]	XhoI - ORF3 - HindIII	ORF3-XhoI-F	GGCC**CTCGAG**GGATGTCAAACAAGATGAAGAGTTTT	ORF3-HindIII-R	CCGG**AAGCTT**CTAGACTCCTACTCTGAA	**pGreenORF3**

Change and addition of tags by mutagenesis procedures

Seq. origin	Amplicon	Forward-primer		Reverse-primer		Resulting product
**Change of N-terminal tag from GFP to His**						
AL V-708[d]	pORF3His	ORF3 C'His F	CATCACCATCACCACTAATCAGCCATACCACATTTGTAGAG	ORF3 C'His R	ATGTCCTCCTCCGACTCCTACTCTGAATCCCTCTTC	**pORF3His (p33^{His})**
**Addition of N-terminal Flag**						
AL V-708[d]	pFlagORF3green	N’Flag F	GATGACGACAAGCCAATGTTCAGGCGAGAATGGTGTTT	N’Flag R	GTCTTTGTAGTCAACAATCCCTTCCCCTACACACAAACAT	pFlagORF3green (^{Flag}p33)

Construction of plasmids expressing deletion variants by mutagenesis procedures

Seq. origin	Amplicon	Forward-primer		Reverse-primer		Resulting product
AL V-708[d]	pORF3green CXC	CXC C'Flag F	GATGACGACAAGTAAAGCGGCCGCGACTC	CXC C'Flag R	GTCTTTGTAGTCTCCGCTCAGTGTCTTTACTAG	pORF3green CXC (p33^{CXCFlag})
AL V-708[d]	pORF3green dCXC	pORF3green dCXC F	ATACTAGTAAAGACACTGAGCGGAGGGG	pORF3green dCXC R	TCCCTCTCCCACCAAGGGTACTCT	pORF3green dCXC (p33$^{\Delta CXC}$)
AL V-708[d]	pORF3green dEx	pORF3green dMid F	ATGTTACCTTTAGTTGTAGGAATAGCCGG	pORF3green dMid R	ACATGTGGCGCTCTGATTGAATAG	pORF3green dEx (p33$^{\Delta Ex}$)
AL V-708[d]	pORF3green dEnv	pORF3green dEnv F	ACAATTGACAATTTAATTGATATGTTACCT	pORF3green dEnv R	AAGATGTTCTACGTGTTGCCCTTTC	pORF3green dEnv (p33$^{\Delta Env}$)
AL V-708[d]	pORF3green dHH	pORF3green dTM F	AAATACTGTAAATGCAAGAAGAAGAGGAC	pORF3green dTM R	ATCAATTAAATTGTCAATTGTGTTTGATTT	pORF3green dHH (p33$^{\Delta HH}$)
AL V-708[d]	pORF3green dHHC	pORF3green dC F	TTAAAGCTTCGAATTCTGCAGTCG	pORF3green dTM R	ATCAATTAAATTGTCAATTGTGTTTGATTT	pORF3green dHHC (p33$^{\Delta HHC}$)
AL V-708[d]	pORF3green dC	pORF3green dC F	TTAAAGCTTCGAATTCTGCAGTCG	pORF3green dC R	GGTCCTCTTCTTCTTGCATTTACAGTATTT	pORF3green dC (p33$^{\Delta C}$)

Construction of plasmids expressing single amino acid residue mutants by mutagenesis procedures

Seq. origin	Amplicon	Forward-primer		Reverse-primer		Resulting product
**Construction of plasmids expressing mutants of specific amino acid variants by mutagenesis procedures**						
AL V-708[d]	pORF3green R46K	ORF3 R46K F	GTGGGAGAGGGAA**A**GGCTGAGAAG	ORF3 46mut R	CAAGGGTACTCTCGCATTCCCTGC	pORF3green R46K (p33^{R46K})
AL V-708[d]	pORF3green R46G	ORF3 R46G F	GTGGGAGAGGGA**G**GGGCTGAGAAG			pORF3green R46G (p33^{R46G})
AL V-708[d]	pORF3green R46E	ORF3 R46E F	GTGGGAGAGGGA**GA**GGCTGAGAAG			pORF3green R46E(p33^{R46E})
Wt 3[e]	pORF3green wt3 K46R	ORF3 K46R F	GTGGGAGAGGGAA**G**GGCTGAGAAG			pORF3green^{wt3} K46R (p33 wt3^{K46R})
Wt 3[e]	pORF3green wt3 K46G	ORF3 K46G F	GTGGGAGAGGGA**G**GGGCTGAGAAG			pORF3green^{wt3} K46G (p33 wt3^{K46G})
Wt 3[e]	pORF3green wt3 K46E	ORF3 K46E F	GTGGGAGAGGGA**GA**GGCTGAGAAG			pORF3green^{wt3} K46E (p33 wt3^{K46E})

a) Template used is rPMCV-pUC57, i.e. full genome sequence of PMCV AL V-708 in pUC57 vector
b) Template used is cDNA synthesized from total RNA extracted from salmon heart tissue originating in field cases
c) Template used is cDNA available in the lab from total RNA extracted from salmon heart tissue sampled from Atlantic salmon experimentally infected with PMCV
d) Template used is pORF3green
e) Template used is pORF3 ^{wt3}green
